# The Degree of Cardiac Remodelling before Overload Relief Triggers Different Transcriptome and miRome Signatures during Reverse Remodelling (RR)—Molecular Signature Differ with the Extent of RR

**DOI:** 10.3390/ijms21249687

**Published:** 2020-12-18

**Authors:** Patrícia G. Rodrigues, Daniela Miranda-Silva, Xidan Li, Cláudia Sousa-Mendes, Ricardo Martins-Ferreira, Zaher Elbeck, Adelino F. Leite-Moreira, Ralph Knöll, Inês Falcão-Pires

**Affiliations:** 1 Cardiovascular Research and Development Center, Faculty of Medicine of the University of Porto, 4200-319 Porto, Portugal; rodrigues13patricia@gmail.com (P.G.R.); daniela.miranda143@gmail.com (D.M.-S.); ccsousam@gmail.com (C.S.-M.); ricardoalexandre_ferreira17@hotmail.com (R.M.-F.); amoreira@med.up.pt (A.F.L.-M.); 2 Department of Medicine, Integrated Cardio Metabolic Centre (ICMC), Heart and Vascular Theme, Karolinska Institutet, 171 77 Stockholm, Sweden; xidan.li@ki.se (X.L.); zaher.elbeck@ki.se (Z.E.); ralph.knoell@ki.se (R.K.); 3 Department of Cardiothoracic Surgery, São João Hospital Center, 4200-319 Porto, Portugal; 4 Bioscience Cardiovascular, Research and Early Development, Cardiovascular, Renal and Metabolism (CVRM), BioPharmaceuticals R&D, AstraZeneca, 431 50 Gothenburg, Sweden

**Keywords:** reverse remodelling, pressure-overload, diastolic dysfunction, myocardial metabolism, fibrosis

## Abstract

This study aims to provide new insights into transcriptome and miRome modifications occurring in cardiac reverse remodelling (RR) upon left ventricle pressure-overload relief in mice. Pressure-overload was established in seven-week-old C57BL/6J-mice by ascending aortic constriction. A debanding (DEB) surgery was performed seven weeks later in half of the banding group (BA). Two weeks later, cardiac function was evaluated through hemodynamics and echocardiography, and the hearts were collected for histology and small/bulk-RNA-sequencing. Pressure-overload relief was confirmed by the normalization of left-ventricle-end-systolic-pressure. DEB animals were separated into two subgroups according to the extent of cardiac remodelling at seven weeks and RR: DEB1 showed an incomplete RR phenotype confirmed by diastolic dysfunction persistence (E/e’ ≥ 16 ms) and increased myocardial fibrosis. At the same time, DEB2 exhibited normal diastolic function and fibrosis, presenting a phenotype closer to myocardial recovery. Nevertheless, both subgroups showed the persistence of cardiomyocytes hypertrophy. Notably, the DEB1 subgroup presented a more severe diastolic dysfunction at the moment of debanding than the DEB2, suggesting a different degree of cardiac remodelling. Transcriptomic and miRomic data, as well as their integrated analysis, revealed significant downregulation in metabolic and hypertrophic related pathways in DEB1 when compared to DEB2 group, including fatty acid β-oxidation, mitochondria L-carnitine shuttle, and nuclear factor of activated T-cells pathways. Moreover, extracellular matrix remodelling, glycan metabolism and inflammation-related pathways were up-regulated in DEB1. The presence of a more severe diastolic dysfunction at the moment of pressure overload-relief on top of cardiac hypertrophy was associated with an incomplete RR. Our transcriptomic approach suggests that a cardiac inflammation, fibrosis, and metabolic-related gene expression dysregulation underlies diastolic dysfunction persistence after pressure-overload relief, despite left ventricular mass regression, as echocardiographically confirmed.

## 1. Introduction

A variety of cardiac pathologies, including ischemic diseases, hypertension, valvular diseases, and genetic forms of cardiomyopathies [1], trigger extensive myocardial remodelling and eventually lead to heart failure (HF). Despite the burden of HF, its prognosis remains unchanged, and therapies targeting specific subtypes remains inefficient as assessed by many HF clinical trials that systematically failed, such as those targeting inflammation, metabolism, or oxidative stress, key players of HF pathophysiology. For more detailed information on HF diagnosis and treatment, please see reference [2].

During cardiac remodelling, the heart geometry and ventricular mass change in parallel with important cellular and molecular modifications, such as cardiomyocyte hypertrophy, excitation-contraction coupling impairment, apoptosis, metabolic disturbances, and inflammation and extracellular matrix (ECM) remodelling. Myocardial maladaptive remodelling is an essential aspect of the disease progression, and its prevention or reversal are desired strategies. Myocardial remodelling can be entirely reversed upon a treatment, while in other cases, reverse remodelling (RR) is incomplete, and the underlying mechanisms remain to be clarified. 

Aortic valve replacement (AVR) is the recurrent treatment for myocardial remodelling associated with long-standing aortic valve stenosis (AS) [3]. An intriguing aspect of AS is the diversity of ventricular responses to the same degree of pressure-overload and overload relief induced by AVR. Cellular and molecular mechanisms underlying the (in)completeness of the RR process in patients after AVR are not yet well understood, mostly due to the impossibility of accessing myocardial tissue and studying its changes during RR. Nevertheless, the degree of myocardial remodelling, the diastolic dysfunction, and the fibrosis before AVR are related to hemodynamic markers of myocardial performance, including end-diastolic pressure and left ventricle ejection fraction (LVEF) [3]. Moreover, excessive myocardial fibrosis, at the moment of AVR surgery, has been associated with impaired recovery of the left ventricle (LV) systolic function, poor long-term outcomes after the treatment [4,5], and correlated with levels of systemic and tissue inflammation markers, such as interleukin-6 (Il-6) and C-reactive protein [6].

Interestingly, the degree of diastolic dysfunction in these patients correlates with the phosphocreatine-to-ATP ratio levels after AVR [7]. During AS remodelling, a metabolic shift from fatty acid towards myocardial glucose utilization has been described [8]. However, the knowledge of metabolic substrate changes after AVR is less understood.

In the last couple of years, current advances in the transcriptional analysis have begun to unravel the underlying dysregulation of the cardiac transcriptome/miRome in the pathogenesis of myocardial remodelling, RR, and HF [9,10]. Regulation of the transcriptome in the heart is the primary determinant of its gene expression signature, phenotype, and function [11]. Also, microRNAs (miRs), non-coding RNA molecules with 22 nucleotides, have been identified as regulators of global gene networks, repressing protein-coding RNAs (messenger RNA, mRNA) translation or causing its degradation. Several studies described the expression of different sets of miRs in cardiac tissue from human HF patients and HF mouse models [12], controlling essential functions in all cells relevant to the cardiovascular system such as endothelial cells, cardiac muscle, smooth muscle, inflammatory cells, and fibroblasts [13].

Murine models have played a significant role in advancing our understanding of the molecular mechanisms involved in the adverse LV remodelling and RR in pressure overload-induced HF [14]. The transcriptome and miRome changes associated with pressure-overload-induced hypertrophy have been extensively described before [15,16]. Thus, we aim to analyze the transcriptome and miRome changes during cardiac RR and identify novel molecular pathways, mechanisms, and targets that might clarify the impact of the remodelling phenotype at pressure overload relief (debanding) and on the extent of the subsequent RR. From a translational perspective, we expect to shed some light on the best timing to perform valve replacement and to clarify the extent to which RR depends on the cardiac remodelling at the moment of AVR.

## 2. Results

### 2.1. In Vivo and In Vitro Characterization of a Cardiac Reverse Remodelling Animal Model

**Figure 1:**
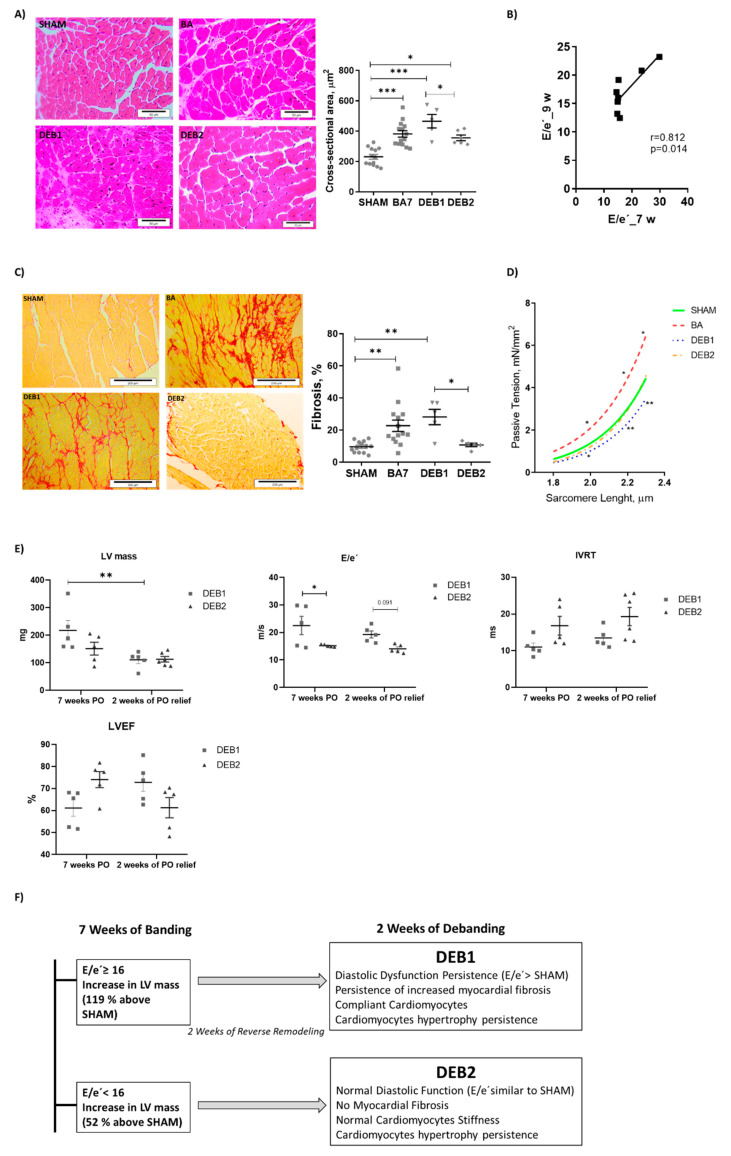
Cardiomyocytes sectional area and representative images of Haematoxylin-eosin-stained sections from (SHAM *n* = 13), BA (*n* = 14), DEB1 (*n* = 5) and DEB2 (*n* = 6) groups (**A**). Pearson correlation between E/e’ before debanding (E/e’ _7w) and after two weeks of debanding (E/e’ _9w) from both DEB1 and DEB2 animals (**B**). Left Ventricular interstitial fibrosis and representative images of Red Sirius-stained sections from SHAM (*n* = 17), BA (*n* = 13), DEB1 (*n* = 4) and DEB2 (*n* = 6) groups (**C**). Cardiomyocytes stiffness assessed by passive tension-sarcomere length relation (number of cells: SHAM, *n* = 49; BA, *n* = 51; DEB1, *n* = 13, DEB2, *n* = 17) (**D**). Echocardiographic characterization of DEB1 and DEB2 subgroups before debanding-induced reverse remodelling (RR) (seven weeks of banding) and after two weeks of cardiac RR (**E**). Summary of phenotypic findings for debanding subgroups (**F**). E/e’, the ratio of mitral peak velocity of the early filling (**E**) to early diastolic mitral annular velocity (e’); IVRT, isovolumetric relaxation time; LVEF, left ventricle ejection fraction; LV mass, left ventricle mass; PO, pressure overload. Values are represented as mean ± SEM. One-Way-ANOVA followed by Holm Sidak’s test for variable fibrosis and CSA. Kruskal-Wallis test for sarcomere length relation. Mixed-effects analysis for echocardiographic variables over time in groups DEB1 and DEB2. vs. SHAM *, *p* < 0.05; **, *p* < 0.01; ***, *p* < 0.001.

Ascending aorta constriction imposes increased LV afterload, as assessed by left ventricle end-systolic pressure (LVESP), and triggers ventricular remodelling (Table 1). Accordingly, the aorta constriction group (banding group, BA) shows marked hypertrophy, displaying increased LV + septum weight (Table 1) and LV cardiomyocytes sectional area (BA vs. SHAM: 381 ± 21 vs. 231 ± 16, *p* < 0.001, Figure 1A. Hypertrophy was accompanied by an increased ratio of E/e’, index of ventricular filling pressure, and increased left ventricular end-diastolic pressure (LVEDP) (Table 1; Figure S1), suggesting the presence of diastolic dysfunction. The LVEF and end-diastolic volume (EDV) were both unchanged seven weeks after banding (Table 1). In BA group, diastolic dysfunction is possibly explain the by increased fibrosis (BA vs. SHAM: 22.67 ± 3.59 vs. 9.59 ± 0.98%, *p* = 0.006) and increased cardiomyocyte stiffness, assessed by higher passive tension-sarcomere length relation (Figure 1C,D). Also, electron microscopy (ME) revealed sarcomere disarray and decreased mitochondria in group BA compared to SHAM (Figure S2B–G).

Two weeks after debanding, echocardiography evaluation revealed the existence of two subgroups: (1) DEB1 subgroup presenting persistent LV hypertrophy (LV + septum weight: DEB1 vs. SHAM, *p* = 0.069, Table 1; and increased cardiomyocytes sectional area: DEB1 vs. SHAM: 465 ± 44 vs. 231 ± 16, *p* < 0.001; DEB1 vs. BA, *p* = 0.060; Figure 1A) and diastolic dysfunction, as assessed by increased E/e’ (Table 1 and Figure 1E) and a trend towards a decrease in isovolumetric relaxation time (IVRT) (vs SHAM, *p* = 0.069, Table 1 and Figure 1E); and (2) DEB2 subgroup, where LV hypertrophy and diastolic function reverted towards SHAM values (Table 1 and Figure 1E). Though, we observed a persistent increase in cardiomyocytes sectional area in DEB 2 (DEB2 vs. SHAM: 356 ± 18 vs. 231 ± 16, *p* = 0.005; DEB2 vs. BA, *p* = 0.472; DEB1 vs. DEB2, *p* = 0.048, Figure 1A), despite normalization of LV mass and weight to SHAM values. Cardiac RR was triggered by a significant decrease of LV afterload (LVESP, ~20%) in both subgroups, as assessed at the final hemodynamic evaluation (Table 1). Despite a similar decrease of LVEDP in both subgroups, DEB1 presented higher E/e’ and a trend towards increased LAA (*p* = 0.298) when compared to SHAM (Table 1). The EDV and lungs weight did not change in both subgroups two weeks after debanding, although lung weight was tendentially higher in DEB1 (Table 1).

Notably, the differences between DEB1 and DEB2 regarding the extent of RR can be ascribed to the myocardial remodelling phenotype immediately before debanding (7 weeks of pressure overload) as assessed by echocardiography (Figure 1E). The E/e’ ratio and IVRT were respectively a tend to increased and decreased at the time of debanding (7 weeks pressure overload) in DEB1 when compared to DEB2. The former remained unchanged throughout the RR process (two weeks of pressure overload relief), as assessed by the significant correlation between E/e’ at the time of debanding (E/e’ _7w) and E/e’ after RR (E/e’ _9w: R = 0.812, *p* = 0.014, Figure 1B). Also, LV mass showed a trend towards an increase in the DEB1 subgroup compared to DEB2 (*p* = 0.134, Figure 1E). Altogether, these findings corroborate the idea of a more severe cardiac remodelling in the DEB1 subgroup (Figure 1E) at the time of debanding that underlies its subsequent “incomplete” RR. In humans, the degree of cardiac remodelling is diverse despite similar aorta gradient and valve dysfunction, and the severity of the diastolic cardiac dysfunction at the moment of AVR, together with other factors, influence the process of cardiac RR after AVR (incomplete vs. complete cardiac RR) [17].

Cardiomyocytes stiffness normalized in both DEB1 and DEB2 groups (Figure 1D), suggesting that stiffness was not a contributing factor to the diastolic dysfunction observed in DEB1 after pressure overload relief. Accordingly, fibrosis persisted increased in DEB1 while it normalized in DEB2 (DEB1 vs. SHAM: 28.14 ± 4.77 vs. 9.59 ± 0.98%, *p* = 0.005; DEB1 vs. BA: *p* = 0.477; DEB2 vs. SHAM: 10.72 ± 1.11 vs. 11.17 ± 1.19%, *p* = 0.823; DEB2 vs. BA: *p* = 0.063; DEB1 vs. DEB2, *p* = 0.026, Figure 1C). The number of mitochondria normalized in both DEB1 and DEB2, but the mitochondria area was increased in the former (Figure S2C–G). Moreover, mitochondria from the DEB1 group presented structural modifications that suggest mitochondrial impairment, such as loss of cristae density and vacuole-like structures (Figure S2E).

### 2.2. Transcriptome Profile in Experimental Cardiac Reverse Remodelling

**Figure 2:**
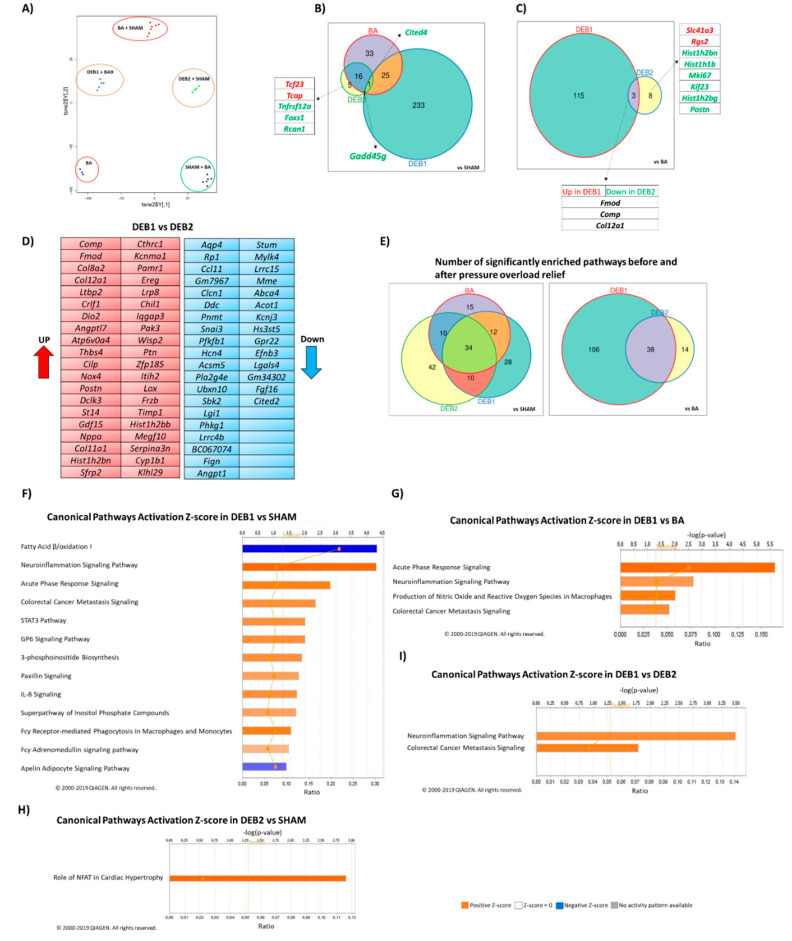
Two-dimensional t-SNEi projection of all initial LV samples (SHAM, BA, and DEB subgroups) and samples from animals with nine weeks of banding (Table S1) (**A**). Differentially expressed transcripts in BA, DEB1, and DEB2 groups when compared to SHAM (**B**), in DEB1 and DEB2 when compared to BA group (**C**), and the top expressed transcripts between the two debanding subgroups (**D**). When the number of differentially expressed transcripts was superior to 15, we referred to it in supplementary data as an excel file format (Data S1). The cut-off for differentially expressed transcripts was set for a false discovery rate (FDR) of ≤0.05, using Benjamini-Hochberg calculation, and a Log2FC ≤ −1 (blue transcripts) or Log2FC ≥ 1 (red transcripts). Venn diagram displaying the number of common and exclusive pathways between the four groups (**E**). Canonical pathways activation Z-score analysis in DEB1 vs. SHAM (**F**), DEB1 vs. BA (**G**), DEB2 vs. SHAM (**H**), and DEB1 vs. DEB2 (**I**). Pathways with Z-score ≥1.5 were predicted to be active (orange bars), and pathways with Z-score ≤1.5 were predicted to be inhibited (blue bar). The threshold for significance was –Log(*p*-value) ≥1.3 (Threshold line) (Fisher’s Exact Test *p*-value). The orange line represents the ratio between Up-regulated and Down-regulated transcripts. Analysis performed using Ingenuity Pathways Analysis Software, Qiagen.

To comprehensively assess the effects of pressure-overload and its relief in the transcriptome, we performed total RNA-Seq using representative LV samples from the different groups collected after terminal hemodynamic. Two-dimensional t-SNEi projection of all initial LV samples (Figure 2A) showed a weak separation between SHAM and BA samples (3 clusters), despite a ~30% increase of afterload (LVESP) and ~50% increase in LV mass. Regarding the DEB group, as mentioned above, we observed two distinct transcriptomic signatures as the samples were selected from subgroups previously created, considering their morpho functional phenotype (DEB1 and DEB2). While DEB2 animals presented an intermediate transcriptomic profile between SHAM and BA, DEB1 revealed a unique transcriptome fingerprint, distinct from all other groups that further confirmed the existence of two subgroups of DEB at the transcriptomic level. Interestingly, DEB1 samples cluster with LV samples from animals with nine weeks of pressure-overload, which showed a more severe phenotype (Table S1).

#### 2.2.1. Pressure Overload-Induced Remodelling (BA vs. SHAM)

In total, we found 75 differentially expressed transcripts, 20 down-regulated and 55 up-regulated, in BA compared to SHAM (Data S1). The lower number of genes is a consequence of high variability in the transcriptome profiles of BA samples. On the one hand, the BA group showed up-regulation of transcripts associated to cardiac fetal gene program in the heart (e.g., *Myl7* and *Myl4*), ECM and cell to cell communication (e.g., *Comp*, *Fmod*, *Ltbp2*, *Thbs4*, *Col11a2* and *Col8a2*), oxidative stress and metabolism (e.g., *Nox4*), angiogenesis (e.g., *Angptl7* and *Vegfd*), protein control process (e.g., *Rhobtb1* and *Dtl*), and circadian clock and clock-controlled transcripts (e.g., *Tef*, *Per2*, and *Per3*) (Data S1). On the other hand, the BA group also showed down-regulation of transcripts related to ECM and inflammation (e.g., *Spon2*, *Mmp3*, *Has2*, and *Nfil3*), clock-controlled, and metabolism (e.g., *Arntl* and *Npas2*) and cell cycle (e.g., *Cdkn1a*) related transcripts.

#### 2.2.2. Debanding-Induced Reverse Remodelling (DEB1 and DEB2 vs. BA)

After pressure-overload relief, and as expected from its more dramatic phenotype, the DEB1 group presented a higher number of differentially expressed transcripts than the DEB2 when compared to SHAM (Data S1). Figure 2B,C display the shared and exclusive differentially expressed transcripts between the four groups and between DEB1 and DEB2, respectively. Compared to SHAM, the two subgroups only share two transcripts, *Gadd45g* and *Cited4*; both downregulated (Figure 2B; Data S1). Interestingly, the former, a growth factor associated with MAPK, FoxO, and p53 signaling, was exclusively dysregulated in both DEB groups, suggesting a specific role of *Gadd45g* in the process of RR. Of note, the ECM remodelling related transcripts, *Comp*, *Fmod*, and *Col12a1*, had different expression levels in debanding subgroups than the BA group. In DEB1, they remained up-regulated, while in DEB2, they decreased after debanding (Figure 2C; Supplement S1).

The top dysregulated transcripts in DEB1, when compared to DEB2, (Figure 2D) are associated with: (a) ECM remodelling, including the *Comp*, *Fmod*, *Ltbp2*, *Thbs4*, *Cilp*, *Postn*, *Timp1*, *Angptl7*, and *Lox*; (b) oxidative stress, inflammation, and angiogenesis, including *Nox4*, *Crlf1*, *Chil1*, *Gdf15*, *Sfrp2*, and *Angpt1*; (c) cardiomyocyte injury-related genes, such as *Nppa* and *Nppb*; (d) and metabolism, like *Ddc*, *Acsm5*, *Pla2g4e*, and *Acot1* (Data S1).

### 2.3. Enrichment Analysis: Candidate Pathways

**Figure 3:**
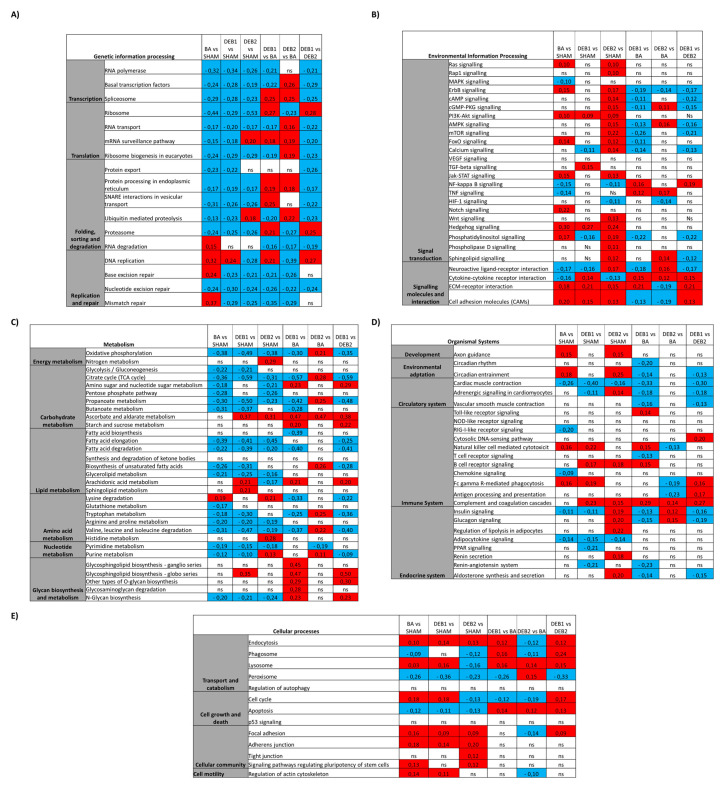
Gene-term Enrichment Pathways analysis. Tables show pathways related to genetic information processing (**A**), environmental information process (**B**), metabolism (**C**), organismal systems (**D**), and cellular processes (**E**) for SHAM vs. BA, DEB1 and DEB2; BA vs. DEB1 and DEB2; and DEB1 vs. DEB2. The MES value, which represents the level of a pathway, is showed for each comparison. A positive MES value (red squares) indicates higher gene enrichment (up-regulation), whereas a negative MES value (blue squares) indicates lower gene enrichment (down-regulation) for each pathway.

Figure 2F–I and Figure 3A–E show the candidate pathways most dysregulated in all groups. For this purpose, we performed two different analyses: (a) IPA activation Z-score that leverages knowledge about the direction of effects, activation/inhibition of a pathway (Figure 2F–I, Data S2 and Figure S8 and (b) GSEA, based on the intersection of the differentially expressed gene with sets of genes that were associated with a pathway (Figure 3A–E).

#### 2.3.1. Pressure Overload-Induced Remodelling Fingerprint (BA vs. SHAM)

After seven weeks of pressure-overload, gene enrichment analysis showed 71 pathways significantly enriched (Figure 2E). We found genetic information processing related pathways, namely DNA replication, mismatch repair, base excision repair and cell cycle, suggesting activation of cell growth and proliferation-related genes on the top of the most enriched pathways. Activation Z-score was significantly positive for cardiac hypertrophy signaling, calcium signaling, and phospholipase C signaling, underlying hypertrophy in the BA group (Figure S3; Data S2). Moreover, inflammation and ECM remodelling related pathways were up-enriched (Figure 3B) and predicted to be activated in the BA group (Figure S3; Data S2). Curiously, we observed down-enrichment of protein quality control related pathways, including ubiquitin-mediated proteolysis and proteasome (Figure 3E) and metabolic pathways, including fatty acid elongation, oxidative phosphorylation, and TCA cycle (Figure 3C).

#### 2.3.2. “Incomplete” Reverse Remodelling Fingerprint (DEB1)

Two weeks after pressure overload relief, Citrate cycle (TCA), Propanoate and butanoate metabolism, and Oxidative phosphorylation pathways were the topmost down-regulated pathways in DEB1 when compared to SHAM and BA (Figure 3C). Fatty acid metabolism, Glycolysis/gluconeogenesis, and PPAR signaling were down enriched compared to SHAM (Figure 3C). Accordingly, in IPA analysis, the Fatty acid β-oxidation canonical pathway was predicted to be inhibited in DEB1 (Z-score = −2.646) (Figure 2F; Data S2). Amino acid metabolism-related pathways were down-enriched in DEB1, including (a) lysine, essential for fatty acids transportation into mitochondria; (b) tryptophan, and the branch chain amino acids (BCCA) valine, leucine, and isoleucine degradation (Figure 3C), suggesting impairment of other cardiac metabolic substrate pathways. Glycan biosynthesis and metabolism and ECM-receptor interaction pathways were up-enriched in DEB1 compared to SHAM and BA, which might underly the persistence of fibrosis. Accordingly, the Apelin cardiac fibroblast signaling pathway, essential for inhibiting cardiac fibroblast activation and differentiation into myofibroblasts, was predicted to be inhibited (Z-score = −1.633; Figure 2F). Several inflammatory and immunity-related pathways were activated in DEB1 when compared to SHAM (Figure 2F) and BA (Figure 2G) (Data S2), including STAT3, neuro-inflammation, and acute phase response signaling pathways. In contrast, the GP6 and IL-8 signaling persisted activated in this subgroup. IPA upstream analysis prediction, that aims to unravel master regulators in the set of differentially expressed genes (DEGs) list, predicted Tgf-β1 as the principal regulatory molecule in the DEB1 subgroup when compared with SHAM and DEB2 groups (SHAM: Activation Z-score = 5.960; *p*-value < 0.001; DEB2: Activation Z-score = 4.745; *p*-value < 0.001; Figure S7), highlighting its potential role in “incomplete” RR.

#### 2.3.3. “Complete” Reverse Remodelling Fingerprint (DEB2)

DEB2 showed a significant up-enrichment in Oxidative phosphorylation, TCA, and Biosynthesis of unsaturated fatty acids when compared to BA, suggesting metabolic energy and metabolism improvement, but incomplete, since TCA and oxidative phosphorylation remained down-enriched compared to SHAM (Figure 3C). Similarly to DEB1, Ascorbate and aldarate metabolism were at the top of the up-enriched pathway in DEB2, when compared to SHAM and BA, indicating changes in metabolic substrate utilization in both subgroups of debanding in comparison to healthy and hypertrophied hearts, respectively (Figure 3C).

Interestingly, Circadian entrainment, an essential regulator of cardiovascular physiology and disease, persisted up-enriched in DEB2 compared to SHAM (Figure 3C). Protein quality control pathways were on the top of dysregulated pathways, namely up-enrichment of Ubiquitin-mediated proteolysis and downenrichment of proteasome in DEB2 compared to both BA and SHAM. Importantly, DNA replication was the most down-enriched pathway when compared to BA and aldosterone-related pathways was the most up-enriched in DEB2 when compared to SHAM, suggesting, on the one hand, cell growth inhibition and, on the other hand, cardiac myocyte hypertrophy stimulation (Figure 3A,D). Accordingly, the nuclear factor of activated T-cells (NFAT) signaling in cardiomyocytes was predicted to be active in DEB2 when compared to SHAM (Figure 2H).

#### 2.3.4. “Incomplete” vs. “Complete” Reverse Remodelling (DEB1 vs. DEB2)

Citrate cycle pathway, oxidative phosphorylation, propanoate metabolism, and amino acid metabolism-related pathways were in the most down-enriched pathways in DEB1 when compared to DEB2. Nucleotide excision repair and mRNA surveillance pathway were down-enriched in DEB1, whereas DNA replication was up-enriched, implying genetic information processing differences between debanding subgroups. Ubiquitin mediated proteolysis and mTOR signaling were down-enriched in DEB1, while proteasome was up-enriched (Figure 3A). As expected, the ECM-receptor interaction pathway and the Glycosphingolipid biosynthesis pathway were up-enriched in DEB1 compared to DEB2. Interestingly, phagosome related genes were up-enriched, suggesting recycling and “cleaning” of cellular components in DEB1. Moreover, IPA analysis predicted activation of Neuroinflammation signaling in DEB1 compared to DEB2 (Figure 2I; Data S2). Genes associated with ECM remodelling (e.g., *Tgf-β3* and *Mmp*) and inflammation (e.g., *Tlr 4* and *Tlr 13*) related to Colorectal Cancer Metastasis Signalling were predicted to be active in DEB1 when compared to DEB2 (Figure 2I; Data S2). IPA upstream analysis predicted that the cytokine Tnf-α has an upstream regulator in DEB1 compared to DEB2 (Activation Z-score = 2.570; *p*-value < 0.001).

### 2.4. MiRome Profile in Experimental Cardiac Reverse Remodelling

**Figure 4:**
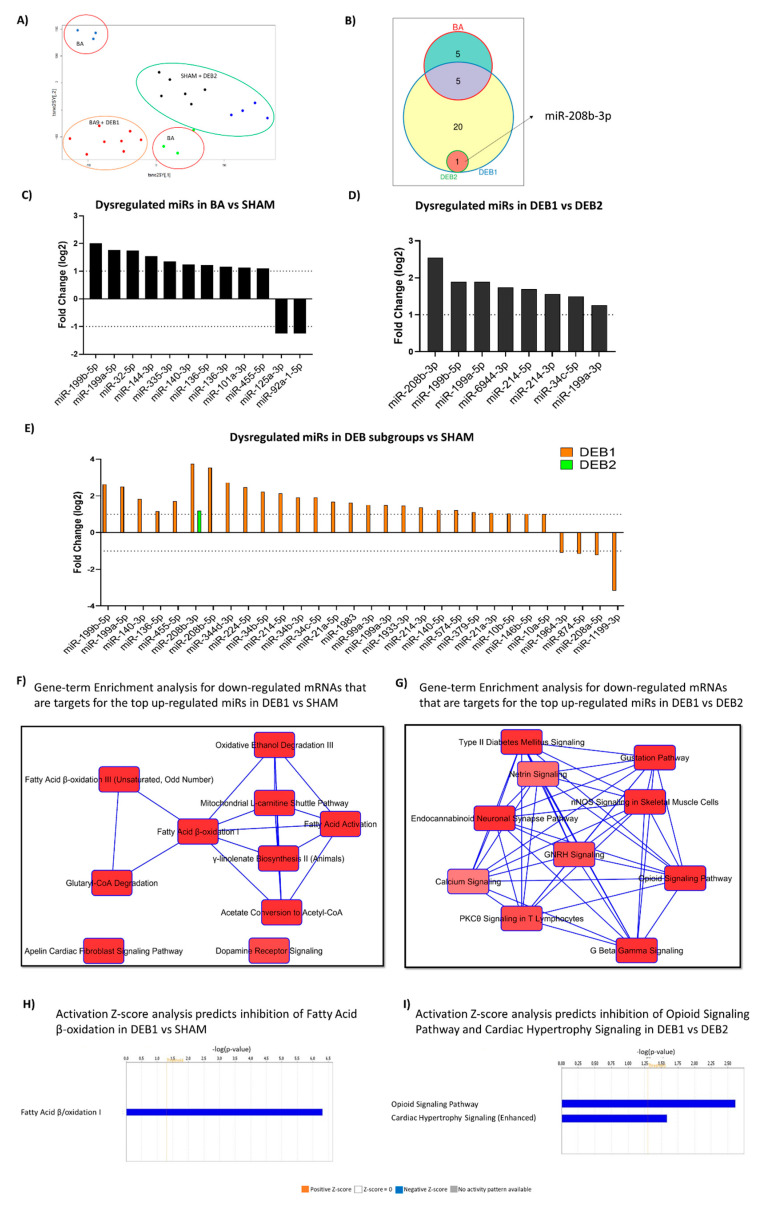
Two-dimensional t-SNEi projection of all initial LV samples, including samples from animals with aorta constriction for nine weeks (Table S1) (**A**). Venn diagram with exclusively and common miRs between BA, DEB1, and DEB2 when compared to SHAM (**B**). Log2FC for miRs found dysregulated in BA vs. SHAM (**C**), in DEB1 vs. DEB2 (**D**), and DEB1 and DEB2 subgroups vs. SHAM (**E**). The cut-off for differentially expressed miRs was set for a false discovery rate (FDR) ≤ 0.05 and |Log2FC| ≥ 1. Integrative mRNA/miR expression profiling shows the overlapping of the ten most enriched canonical pathways found in DEB1 compared to SHAM (**F**) and DEB2 (**G**). Since the primary biological role of miR is mRNA translation inhibition or degradation, the data set was filtered to show only relationships with miR up-regulated (Log2FC ≥ 1) and mRNA downregulated (Log2FC ≤ −0.5). The network of overlapping canonical pathways shows each pathway as a single “node” colored proportionally to the Fisher’s Exact Test *p*-value, where brighter red is equal to more significant. A line connects any two pathways when at least one data set is shared between them. Activation Z-score analysis results are showed for each comparison (**H**,**I**). Ingenuity pathway analysis software allowed to combine data set from miR and mRNA seq, aiming to discover a potential relationship between these two biological molecules and their role in reverse cardiac remodelling.

To comprehensively assess the effects of pressure-overload in miR expression, we performed Small-RNA Seq using an enriched miRs fraction (Table S2). Two-dimensional t-SNEi projection of all initial LV samples (Figure 4A) showed weak separation between BA and SHAM samples. The separation between the DEB subgroups was not as pronounced as in transcriptomic analysis.

Despite variability in BA miRome profiles (spread over 2 clusters), we observed 29 dysregulated miRs compared to SHAM (Figure 4C; Data S3). The DEB1 subgroup showed the highest number of dysregulated miRs compared to SHAM (Figure 4B–E; Data S3). The subgroup DEB2 showed the up-regulation of the myomiR-208b-3p exclusively compared to SHAM (Figure 4E; Data S3). Both DEB subgroups did not show dysregulated miRs when compared to the BA group. However, compared to each other, eight miRs appeared up-regulated in DEB1, including myomiR 208b-3p (Figure 4D). The increased expression of miR-214-3p was re-confirmed by RT-PCR analysis (Figure S4), re-confirming an essential role of this miR in the RR associated with diastolic dysfunction and fibrosis persistence. Both miR-199a-3p and miR-34c-5p showed a trend towards high expression in DEB1 compared to DEB2 (Figure S4).

### 2.5. Integrative mRNA/miR Expression Profiling

IPA microRNA target filter analysis allowed the combination of data sets from miR and mRNA sequencing, aiming to discover a potential relationship between these two biological molecules and their possible role in cardiac RR (Figure 4F–I). Information about the miR and its predicted mRNA targets and pathway analysis are described in Data S4 and Data S5, respectively.

#### 2.5.1. Pressure Overload-Induced Remodelling Fingerprint (BA vs. SHAM)

Eight out of 10 miRs have as predicted targets 25 mRNAs significantly dysregulated in the BA group compared to SHAM (Data S4). The microRNAs 32-5p and 199b-5p have as predicted targets 7 and 6 mRNAs, respectively, and the most down-regulated targets within the 25 targets were *Ddit4*, *Eln*, *Has2*, *Nfil3*, *Npas2*, and *Cdkn1a* (Figure S5; Data S4).

Pathway analysis of the 25 dysregulated targets predicted 16 pathways to be significantly enriched in the BA group, including those associated with cellular growth, stress, metabolism, nervous system, and inflammation (Figure S6; Data S5), all features known to be modulated in the context of pressure-overload remodelling.

#### 2.5.2. “Incomplete” Reverse Remodelling Fingerprint (DEB1 vs. SHAM)

**Figure 5:**
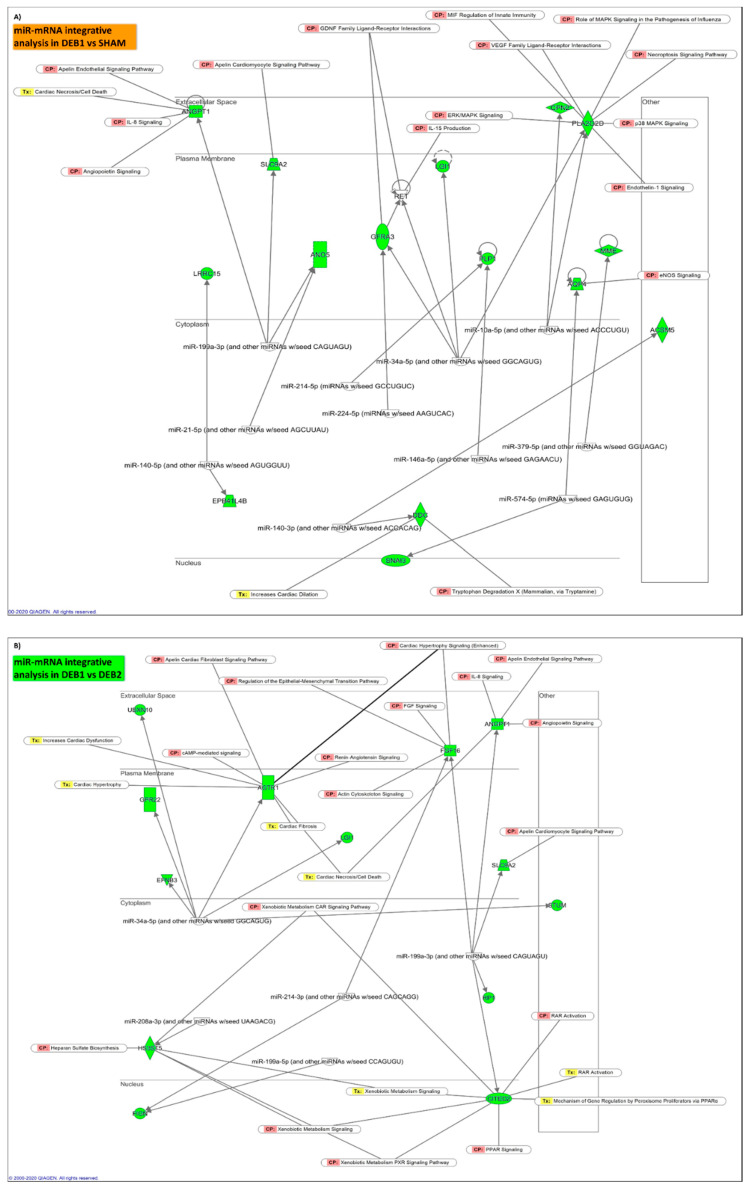
Integrative analysis connections between top down-regulated mRNA (in green) and the top-upregulated miRs in DEB1 when compared with SHAM (**A**) and with DEB2 (**B**). The molecules are distributed accordingly to their subcellular location. Analysis performed using Ingenuity pathway analysis software, Qiagen. CP, Canonical Pathway; Tx, Ingenuity Toxic List.

Twenty out of 22 miRs predictively downregulate 111 mRNA in DEB1 (Data S4). MicroRNA 34b-5p was the miR with the highest number of predicted mRNA (30) and was followed by miR-199b-5p with 17 predicted targets (Data S4). The Kelch-like family member 23 (*Klhl23*) transcript, with a role in protein ubiquitination, was predicted to be a target for 6 different miRs dysregulated in DEB1. However, its level of down-regulation was not very evident (Log2FC = −0.608; Data S4). Aquaporin 4 was the most down-regulated mRNA in DEB1 (Log2FC = −2.388) and was a predicted target for miR-574-5p (Figure 5A).

IPA core analysis for the 111 targets predicted 30 pathways to be significantly enriched in DEB1, the majority related to metabolism, corroborating DEGs global candidate pathway analysis (Figure 4F, Data S5). Fatty acid β oxidation I and acetate conversion to acetyl-CoA were the most significantly enriched pathways (Figure 4H), indicating that the inhibition of fatty acid metabolism and modifications at the TCA level in DEB1 are subjected to miR regulation. Tryptophan degradation, alanine degradation, and Biosynthesis and mitochondrial L-carnitine shuttle pathway were also significantly enriched, corroborating the idea of amino acid metabolism and mitochondria function impairment in DEB1 (Figure 4F; Data S5). Curiously, the apelin cardiac fibroblast signaling pathway was significantly enriched (Figure 4F; Data S5), supporting the prediction for inhibition observed in the enrichment pathways analysis and uncovering this pathway’s role in the “incomplete” RR process.

#### 2.5.3. “Incomplete” vs. “Complete” Reverse Remodelling (DEB1 vs. DEB2)

Six out of 8 of dysregulated miRs showed as predicted targets 50 down-regulated mRNAs in DEB1 compared to DEB2 (Data S5). The miR-34c-5p showed the highest number of predicted targets, 21 mRNAs, followed by miR-199b-5p and miR-214-3p with 13 and 12 targets, respectively. The cluster miR-214~miR-199a-5p has been described as a promoter of the metabolic cardiac switch from fatty acid metabolism to glucose metabolism in stress conditions such as pressure overload conditions [12,18]. The miR-214-3p is also an expression regulator of apoptosis, hypertrophy, and fibrosis-related genes [19,20]. The myomiR-208-3p, the most up-regulated miR in DEB1, presented four predicted targets, including the glycosaminoglycan biosynthesis-related transcript Hs3st5 (Figure 5B; Data S4). This miR is highly enriched in cardiomyocytes and regulates the balance between the α- and β-myosin heavy chain expression. Interestingly, it has been associated with cardiac RR in human HF [21,22]. The miR-34c-5p regulates 5 of the top down-regulated transcripts found in DEB1 vs. DEB2 (Figure 5B). The members of miR-34 family are usually upregulated in the heart in response to stress and its inhibition by using a locked nucleic acid (LNA)-modified antimir-34, improved cardiac function in mice with preexisting pressure overload-induced hypertrophy and systolic dysfunction [23].

Pathway analysis revealed 31 significantly enriched pathways associated with 50 targets (Figure 4G, Data S5). Peculiarly, 9 out of 31 enriched pathways were related to nervous system signalling, including the Opioid signalling pathway that was the most significantly enriched and with a negative activation Z-score (Z-score = −2.000), predicting inhibition of this pathway in DEB1 when compared to DEB2 (Figure 4I, Data S5). Per what was found in DEB1 compared to SHAM, fatty acid activation and mitochondrial l-carnitine shuttle pathway also appeared to be significantly enriched in the DEB1 compared to DEB2 (Data S5), pointing out that metabolic and mitochondria derangements may underly the diastolic dysfunction associated with incomplete cardiac RR. Humoral and cellular immune response pathways also appear as enriched pathways, suggesting once more a role for inflammation in the DEB1 phenotype (“incomplete” RR). Remarkably, the cardiac hypertrophy signalling pathway was predicted to be inhibited in DEB1 compared to DEB2 (Figure 4I). This finding corroborates the prediction for activation of the NFAT signalling pathway in DEB2 compared to SHAM (Figure 2H, Data S2).

## 3. Discussion

**Figure 6:**
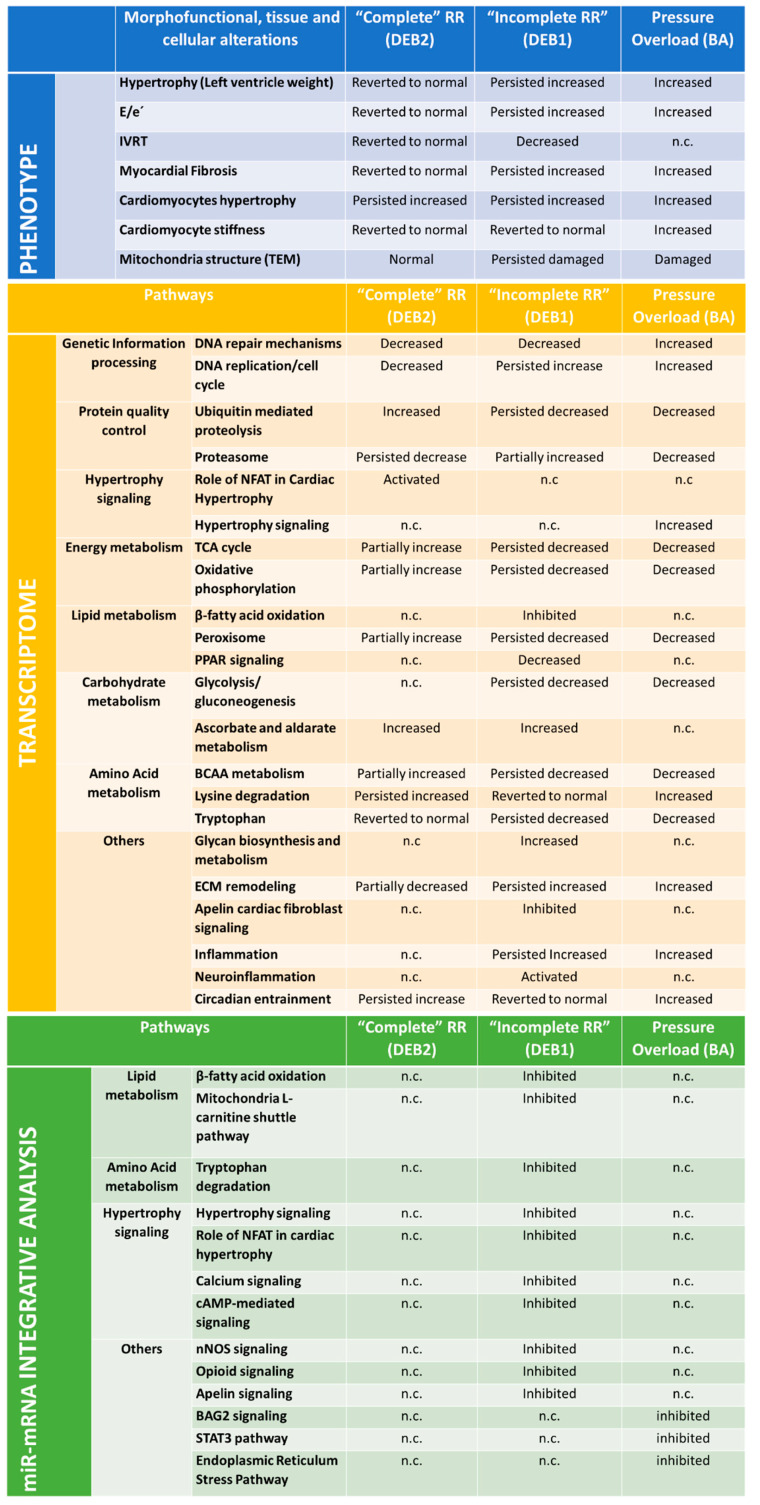
Summary of the most important essential findings after seven weeks of pressure overload (BA group) and after 2 weeks of pressure overload relief (“Incomplete” RR, subgroup DEB1; and “Complete” RR, subgroup DEB2). Table interpretation: ) **“Partially”**, there was a change in DEB subgroups when compared to BA group but without reverting completely to SHAM values; **“Persisted”,** modification observed in BA group didn’t revert in DEB subgroups; **“Reverted to Normal”**, reverted to SHAM values; **“Increased”**, means that it was exclusively increased in the group; **“Decreased”**, means it was decreased solely in the group; **“n.c.”**, no significant or not found dysregulated. IVRT-isovolumetric relaxation Time.

Understanding the global gene networks associated with cardiac remodelling occurring in pressure-overload conditions, such as in AS, provides critical insight underlying the pathophysiology of hypertrophy regression and cardiac disease recovery, including the optimal timing for valve replacement and an unprecedented array of molecular targets for therapeutic intervention. Left ventricle RR represents the sum of a series of integrated biological changes in the genome, transcriptome, and miRome that determine a panoply of ECM, metabolic, inflammation, structural and functional adaptations after a specific intervention (Figure 6).

To the best of our knowledge, this is the first study performing an integrated global mRNA and miR expression profile in mice subjected to reversible banding, revealing novel miR-mRNA networks and new therapeutic targets in the field of cardiac RR. Our results evidence that cardiac remodelling’s phenotype immediately before afterload relief determines the subsequent cardiac RR’s extent. On the one hand, a “complete” cardiac RR presents LV hypertrophy regression, normal diastolic function, fibrosis and cardiomyocytes stiffness, and persistence of cardiomyocytes hypertrophy. On the other hand, an “incomplete” cardiac RR, in which hypertrophy, fibrosis, and diastolic dysfunction persist, while cardiomyocytes become more compliant with increased mitochondria disarrangement. Importantly, we highlight which genes, miRs, and biological pathways are more associated with the two different RR phenotypes. The “complete” RR group of animals was associated with an up-regulation/activation of NFAT signalling, circadian entrainment, and ubiquitin-mediated proteolysis. In contrast, “incomplete” RR presented a potent down-regulation/inhibition of fatty acids oxidation, TCA, oxidative phosphorylation, and apelin-related pathway. Moreover, ECM remodelling, inflammation, and neuroinflammatory pathways were upregulated/activated in this group.

As expected, concentric hypertrophy and diastolic dysfunction, as assessed by E/e’, were patent in the BA group. Typically, the presence of aorta constriction triggers an increase in chamber stiffness and, consequently, a delay in active LV relaxation, which will cause LV diastolic dysfunction, increased filling pressure, and worsening of cardiac function [24]. Our study’s main goal was to describe the diastolic function alterations occurring before and after pressure overload relief. For this reason, we used the E/é ratio as one of the main outputsof the present study since it is a well known surrogate marker of diastolic dysfunction and elevated filling pressures in clinical [25,26] and in pre-clinical [27] setting. However, compared to the existing literature, similar mouse studies have shown systolic dysfunction and LV dilation at earlier time points after aorta constriction [28,29], which might be explained by the differences between strains and mainly by the degree of aortic constriction [29,30]. Several other procedures that aim to induce pressure overload have been described. Most of them provide better ways to standardize the degree of aortic constriction [28], however they lack the possibility of mimicking the natural variability observed in patients. In the present study, the diversity of myocardial response to pressure overload and overload relief was an advantage that allowed to have distinct patterns of cardiac remodelling (hypertrophy and diastolic dysfunction) and consequently RR within our groups, thus better mimicking the clinical scenario. 

The global mRNA and miR expression profile showed the dysregulation of metabolic, inflammatory, hypertrophic, ECM, and adverse remodelling-related pathways seven weeks after the ascending aorta constriction. Other groups have extensively studied the network of genes dysregulated in pressure overload-driven HF, where ECM remodelling, cytoskeleton and inflammation-related pathways also stand as the most relevant pathophysiologic features underlying it [15,31,32]. However, less is known about the mechanisms underlying different cardiac RR phenotypes after pressure overload relief. Therefore, we will focus our discussion on the differences between the “incomplete” and “complete” RR phenotypes, and, whenever adequate, we will compare these groups to the normal phenotype (SHAM group) and the disease one (BA group).

### 3.1. Processes Driving “Incomplete” and “Complete” Reverse Remodelling

An interesting finding of our study is the difference between the extent of cardiac hypertrophy between the “incomplete” RR (DEB1) and “complete” RR (DEB2) groups. While in the former, cardiac hypertrophy persisted increased (like BA), it normalized to SHAM levels in the latter. The transcriptomic analysis suggests that hypertrophic signalling is still present in DEB2 (Data S2), particularly NFAT signalling, usually associated with maladaptive remodelling [33]. This explains in part why cardiomyocytes hypertrophy in LV biopsies from DEB2 persists, despite LV mass and weight normalization. In DEB1, inflammation and ECM remodelling prevailed over to hypertrophy related pathways.

Nevertheless, we still observed the persistence of increased expression of hypertrophy-related genes, such as Myh7, Nppa and Nppb (Data S1), and increased pro-hypertrophic miRs miR-208b-3p and miR-199a-5p in the DEB1 group. Moreover, it is well described that hypertrophy and fibrosis appearance are intrinsically related to each other. For instance, fibroblast growth factor 16 (Fgf16) was down-regulated in DEB1 and was a predicted target for the miR-199b-3p and miR-214-3p, both up-regulated in this subgroup. The absence of fibroblast growth factor 16 has been described as associated with high fibrosis and hypertrophy levels in hearts from mice treated with angiotensin-II [34]. Moreover, the up-enrichment of proteasome and ribosome pathways, together with the ubiquitin-mediated proteolysis down-enrichment, supports the persistence of hypertrophy in DEB1. Similar results were reported in cardiac RR after left ventricle assist device (LVAD) implantation, namely the reduction of ubiquitinated proteins and an increase in proteasome subunits expression [35].

Likewise, we cannot exclude the contribution of post-translational protein modifications in the process of cardiac RR, which can influence the state of activation/inhibition of hypertrophic pathways, as it happens with calcineurin/NFAT signalling [33,36]. In the present study, we did not carry out these types of studies, and therefore, we cannot rule out their involvement in the phenotype observed in different groups.

In agreement with our results, several studies reported that cellular and ECM myocardial changes regress at different rates. In general, myocardial hypertrophy regresses much faster than fibrosis, explaining the incomplete recovery of diastolic dysfunction even when LV mass regress [37,38]. In our study and compared to DEB2 and SHAM group, DEB1 displays a more adverse phenotype imposing an incomplete recovery of diastolic function, as supported by several studies with AVR [39,40]. Notably, after seven weeks of pressure-overload, diastolic dysfunction was more pronounced in DEB1 than in DEB2, as assessed by the higher E/e’. Based on reports from AS patients one year after AVR, the DEB1 phenotype is possibly explained by the existence of replacement fibrosis at debanding time, while DEB2 by a more diffuse pattern of fibrosis accompanied by myocardial function improvement [3]. Intrinsic cardiomyocyte changes also contribute to myocardial remodelling and diastolic dysfunction [41]. After debanding, we observed a normalization of passive tension in cardiomyocytes from both debanding subgroups, suggesting that myocardial fibrosis is the principal contributor for diastolic function impairment predominantly observed in DEB1. Accordingly, we also found a partial regression of cardiomyocytes stiffness and no regression of fibrosis in a similar rat experimental study [36]. In the context of the present study, reduction of cardiomyocyte stiffness can be interpreted as a compensatory response to pressure-overload relief of the “fibrotic” myocardium of the DEB1 subgroup, as previously reported in myocardium from HF patients [42].

Moreover, apelin related pathways consistently appeared to be inhibited in the DEB1 subgroup, in both transcriptomic and miR-mRNA integrative analysis. This protein is an essential inhibitor of cardiac fibroblast activation and differentiation into myofibroblasts [43], highlighting its role for the fibrotic phenotype observed in DEB1. Interestingly, Apelin cardiac fibroblast signalling does not seem to be necessarily involved in fibrosis build-up during pressure-overload, as confirmed by the absence of significant up-enrichment in BA mice.

Glycan biosynthesis-related pathways were exclusively up-enriched pathways in DEB1. Proteoglycans are significant constituents of ECM, and, in the last years, they have been suggested to be important regulators of cardiac remodelling and activation of innate immunity. Several pro-fibrotic glycoproteins, known to be up-regulated in experimental pressure-overload [44] and HF [45,46], were up-regulated in DEB1, including Thbs4, Comp, Omd, Postn, and Fmod. These glycoproteins can act as damage-associated molecular patterns (DAMPs) (endogenous ligands) that will be recognized by the TLRs and Nod-like receptors (NLRs), triggering NF-kβ-dependent inflammatory response [47]. The DEB1 showed up-enrichment of IL-8, ILK, NOD-like, RIG-I, TNF-α, and neuroinflammation signalling pathways, all, possibly, associated with changes in the expression of Tlr4 and Tlr13 and the transcripts Nox4 and Tgf-β in DEB1 (Data S2). The relation between cytokines and chemokines release can lead to a disproportionate synthesis of collagen, fibronectin, and laminin [48], contributing to higher myocardial fibrosis levels and diastolic dysfunction. Enthralling, the critical regulator of ECM remodelling, Tgf-β, was predicted as a master regulator of DEB1. Similar to us, other groups have shown the persistence of dysregulation of genes directly related to integrins/cytoskeleton and matrix/collagen in RR [15,49]. Furthermore, after LVAD implantation, the circulatory levels of inflammation mediators (e.g., increased IL6 and IL8), the macrophage activation (e.g., increased levels of macrophage-1 antigen expression), and the adaptive immune cell deactivation (e.g., increased T-cell apoptosis and defective T-cell response to pathogens) increased and were associated to the worst prognosis of HF patients [50]. Together, these reports suggest that despite pressure-overload relief, insistent activation of various cardiac inflammatory pathways likely precede the fibrotic pattern and, consequently, the unimproved diastolic function observed in DEB1 as we previously demonstrated in an analogous rat model [36].

Another important hallmark of DEB1 was the impairment of metabolic-related pathways, including Fatty acid β-oxidation, Oxidative phosphorylation, TCA, and Mitochondrial L-carnitine shuttle pathway. These results corroborate previous studies showing the interplay between metabolism alterations, mitochondria dysfunction, chronic inflammation, and ECM remodelling [51,52]. In the context of chronic HF, damaged mitochondria can release mitochondrial DNA and oxygen reactive species that will act as DAMPs [52]. DEB1 animals did not show a shift to normal fatty acid metabolism and improvement in oxidative phosphorylation, as others observed [47,53]. Instead, they seem to become even worse, triggering dysregulation of mitochondria-related pathways with marked mitochondria structural changes (Figure S2) that consequently can lead to the recruitment of inflammatory cells, activation of Tgf-β and ultimately cardiac fibrosis [54]. Interestingly, miRs 214-3p and 199a-5p, top up-regulated miRs in DEB1, were described as a cluster capable of targeting myocardial PPAR-δ and impair mitochondrial fatty acid oxidation [18], suggesting a potential role of this cluster in “incomplete” cardiac RR. Moreover, miR-34, well known to be dysregulated in HF experimental models, was also increased in DEB1. It was described that the miR-34 family members have as target mRNAs associated with cardiac metabolism, such as the *Acsl4* [50], important for fatty acid oxidation. Interestingly, miR-34 family inhibition attenuated TAC-induced cardiac remodelling and dysfunction [23].

Amino acids metabolism-related pathways, including BCAA, lysine, and tryptophan degradation, persisted or became dysregulated in DEB1. The decrease in BCAA oxidation’s degradation triggers its accumulation in cardiac tissue, increasing reactive oxygen species-associated damage, and fastening HF progression [55,56] by contributing to the sterile inflammatory response. Interestingly, the levels of BCAA remained decreased in failing hearts after LVAD implantation. Moreover, carnitine deficiency, a collagen precursor, has been associated with fatty acid oxidation impairment [57]. In contrast, by increasing the synthesis of nicotinamide adenine dinucleotide (NAD+), tryptophan regulates ATP production, mitochondria function, DNA repair, and intracellular calcium regulation and cell death [58]. Altogether, our results suggest that the impairment of amino acid metabolism could also contribute to the alterations observed in DEB1 through the increase in oxidative stress, changes in collagen synthesis/degradation ratio, and deviations of immune responses.

Regarding the DEB2 group, we observed signs of partial metabolic improvement, corroborated by increased Oxidative phosphorylation, TCA signalling pathways, and preserved mitochondrial structure compared to BA. In a similar rat RR model, we found that the attenuation of cardiac hypertrophy and oxidative stress allowed myocardial energetics, LV hypertrophy, and diastolic dysfunction to recover, despite the persistence of increased markers of autophagy and mitophagy [36]. Also, in the clinical context, cardiac unloading induced by LVAD [59] or by AVR [7] was associated with a partial reversal of the depressed metabolic gene expression and with a significant improvement of postoperative myocardial phosphocreatine/adenosine triphosphate ratio (PCr/ATP) in parallel with a better LV diastolic function.

Altogether, evidence from our study suggests that the degree of cardiac metabolic and inflammation status recovery seems to parallel diastolic function improvement after cardiac unloading. This strengthens the idea that in addition to diastolic function assessment (by echocardiography), inflammation and cardiac metabolic evaluation (using biomarkers or PET/MRI analysis) should be considered when deciding the best time for valve replacement inferring about RR progression and prognosis.

### 3.2. Limitations

A limitation of this study is that the differential RR observed between the two DEB subgroups can be due to a differential degree of aortic banding in the first place. Ideally, we should have used different needle gauge to induce different pressure overload degrees, cardiac remodelling, and, consequently, distinct cardiac RR (more controlled methodology). Another important limitation was the echocardiographic probe used to detect the aorta velocities and, therefore, the pressure gradients. The probe has a threshold detection velocity of 2.5 m/s (gradient ~25 mmHg), meaning that we were unable to discriminate the animals with aorta velocities >2.5 m/s, explaining in part the variability observed within BA cohort.

Transcriptome and miRome profiling provide an extensive and global characterization of a disease process’s pathophysiology at a specific time-point. In this regard, this profiling does not provide information on the post-translational modifications nor the rate of protein turnover, which significantly impacts the disease progression.

This study did not evaluate the effects of long non-coding RNAs and epigenetic modifications, which have been shown to impact cardiac remodelling and, eventually, cardiac RR significantly. Finally, although cardiomyocytes represent the most significant cardiac cell population, the heart comprises many different cell types, whose contribution to the transcriptomic profile we cannot exclude.

## 4. Materials and Methods

### 4.1. Ethics and Animal Care

This work was conducted in healthy C57B1/J6 mice (JAX stock, #000664, Charles River, Sant Cugat del Vallès, Spain, *n* = 44). Animal experiments complied with the Guide for the Care and Use of Laboratory Animals (NIH Publication no. 85–23, revised 2011) and the Portuguese law on animal welfare (DL 129/92, DL 197/96; P 1131/97). The experimental protocol (018833) was approved by the Portuguese local authority, Direcão-Geral de Alimentacão e Veterinária (DGVA) (4 July 2013). Seven-week-old male C57B1/J6 mice were used and kept in appropriate cages, with a regular 12/12H light cycle environment at a temperature of 22 °C and 60% humidity with access to a standard diet ad libitum.

### 4.2. Animal Model of Reverse Remodelling

Standardized ascending aortic banding/debanding was performed through a left-sided, muscle-saving thoracotomy under a dissecting microscope (50 Zeiss, Oberkochen, Germany) [29]. Randomly the animals were anaesthetized by inhalation of a mixture of oxygen and Sevoflurane (8% and 2.5–3% for induction and maintenance, respectively; TOPO Small Animal Ventilator, Kent Scientific Inc, Torrington, CT, USA). After a small incision between the 2nd and 3rd intercostal space, the aorta was dissected and exposed, and a 26-gauge blunted needle was placed parallel to the aorta to induce aortic constriction. A ligature (7–0 polypropylene) was firmly tied around both the aorta and the needle, and the latter was quickly removed (Banding group, BA). Sham mice underwent the same procedure, but the suture was kept loose (SHAM). The thorax was closed, and the animal recovered with appropriate analgesia (Buprenorphine, 0.1 mg·kg^−1^, twice daily during two days after surgery). After 7 weeks, a second surgery was made in ~half of the BA animals to remove the suture, debanding group (DEB). The chest was opened at the same place as the first surgery. The banding site was identified, and the ligature and aorta fibrous tissue were dissected. Randomly, SHAM animals were also subjected to debanding surgery (SHAM_DEB_) to remove loose ligature. The BA animals with aortic velocity below 2.5 m/s seven weeks after aorta constriction were excluded from the study. The debanding surgery was performed using the same anaesthesia and analgesia regiment of the banding surgery.

### 4.3. Animals’ Euthanasia

**Figure 7:**
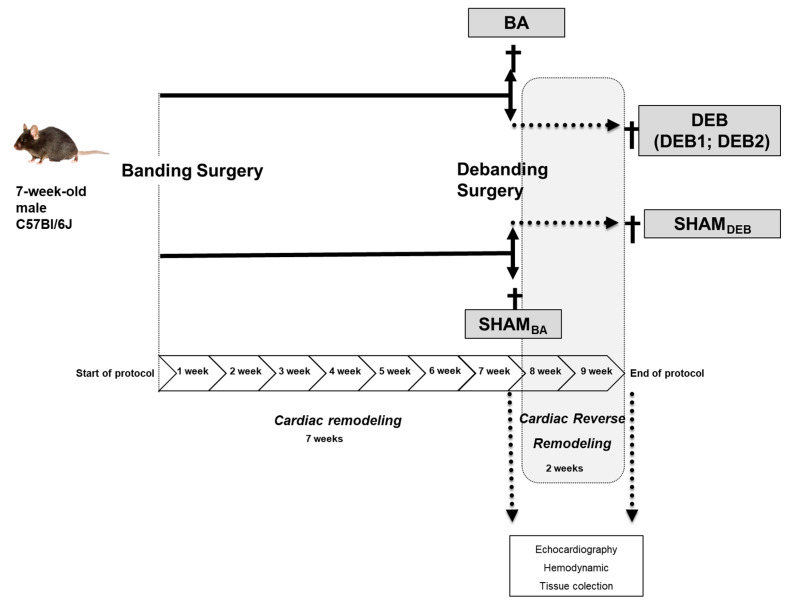
Study design. Seven-week-old C57Bl/6J mice were subjected to ascending aorta constriction (BA group) or a loose suture around the aorta (SHAMBA). After seven weeks, a second surgery was performed in half of BA and SHAM animals to remove the ascending aorta constriction or loose suture (debanding surgery). After two weeks of reverse remodelling, echocardiography and hemodynamic evaluation were performed, followed by animal termination (DEB and SHAM_DEB_ groups). Echocardiographic assessment of the DEB group unveiled the existence of two DEB subgroups with distinct degrees of cardiac dysfunction, namely increased LV mass and severe diastolic dysfunction (DEB1 subgroup) and a second group with increased LV mass but normal diastolic dysfunction (DEB2 subgroup).

Mice were anaesthetized by inhalation of a mixture with oxygen and Sevoflurane (8% and 2.5–3% for induction and maintenance, respectively) inside vented containers and titrated to avoid the toe pinch reflex. Subsequently, after the hemodynamics procedure, the mice were sacrificed at seven weeks after aortic banding (BA and SHAM_BA_) or two weeks after the debanding procedure (DEB and SHAM_DEB_). After hemodynamics, the heart was excised, subdivided into sections, and collected for future studies: (1) fibrosis was quantified using interventricular septum (IVS) samples; (2) myofilamentary diastolic function was assessed using cardiomyocytes isolated from frozen apex samples and (3) Bulk RNA and small sequencing and transmission electron micrographs were carried out using LV posterior wall (LVPW) frozen samples. Our protocol design resulted in 2 SHAM groups: one was subjected to an aortic loose-ligation (SHAM_BA_), and the other was subjected to the aortic loose-ligation and its removal (SHAM_DEB_) as depicted in Figure 7. We have initially handled these two groups separately. However, for each experiment, we found no significant differences between these groups. Therefore, we grouped them as a single group, henceforth named SHAM.

### 4.4. Echocardiography

The cardiac function and structure were evaluated seven weeks after the aortic banding procedure and two weeks after debanding. Briefly, the animals were anaesthetized by inhalation of a mixture of oxygen and Sevoflurane (8% and 2.5–3% for induction and maintenance, respectively) and placed in the left lateral decubitus position of a heating pad (38 °C). Echocardiography was carried out using a linear 15-MHz ultrasound probe equipped with tissue Doppler technology (Sequoia 15L8W) and using an echocardiograph from Siemens Acuson Sequoia C512 (Malvern, PA, USA). Noninvasive evaluation of the pressure gradient across ascending aorta constriction was performed by Doppler echocardiography. The efficacy of debanding was verified by two-dimensional images showing the absence of ascending aorta constriction (Figure S1). Two-dimensional guided M-mode tracings were made for measurements of the intraventricular septum (IVS), LV diameter (LVD), and left ventricular posterior wall (LVPW) in diastole and systole. The mitral peak velocity of the early filling (E wave) was recorded using an apical 4-chamber view (Figure S1). The early diastolic mitral annular velocity (e’ wave) was recorded using pulsed-TDI and apical 4-chamber view (Figure S1). The left atrium area (LAA) was recorded by a 2D image using an apical 4-chamber view. Left ventricular ejection fraction was calculated using the cube method according to the formula: LVEF = [(LVDd3 − LVDs3)/LVDd3] × 100. BA animals with aortic velocity below 2.5 m/s, 7 weeks after aorta constriction, were excluded from the study.

### 4.5. Hemodynamic

All animals were anaesthetized with a mixture of oxygen and Sevoflurane (8% and 2.5–3% for induction and maintenance, respectively) and mechanically ventilated. Under surgical microscopy visualization, the internal jugular vein was catheterized for saline administration. A pressure catheter (PVR-1035; Millar Instruments, Houston, TX, USA) was inserted through the apex and positioned along the long axis of the LV cavity. After 30 min of stabilization, hemodynamic parameters were recorded and analyzed off-line.

### 4.6. Cardiomyocyte Sectional Area and Extracellular Matrix Alterations Evaluation

Myocardial samples were fixated, sliced in serial sections (3 μm thick), and stained by Hematoxylin-eosin and Pico Sirius Red for assessing cross-sectional area (average of 60 orbicular cardiomyocytes per animal) and the percentage of interstitial fibrosis (average of 8 representative fields), respectively. The formula used to calculate the percentage of interstitial fibrosis was ((% Fibrosis)/(%Fibrosis + %Tissue)) * 100.

### 4.7. Isolated Cardiomyocyte Function Evaluation

Force measurements were performed in single permeabilized cardiomyocytes from LV biopsies from all groups as described previously [60]. The diastolic function at the myofilament level (cardiomyocytes’ stiffness) was detected by constructing a passive tension/sarcomere length relation curve. Briefly, myocardial samples were mechanically disrupted and permeabilized using 0.2% Triton X-100. Single cardiomyocytes were attached between a force transducer (Model 403A, Aurora Scientific Inc., Aurora, ON, Canada) and a motor (Model HSVL 315C-I, Aurora Scientific Inc., Aurora, ON, Canada). Cell passive tension/sarcomere length relation was obtained by stretching each cell until 2.3 µm of sarcomere length in a relaxing solution. On average, 30 cardiomyocytes were measured per group.

### 4.8. Electron Microscopy

After tissue harvest, LV samples were immediately fixed in 2.5% glutaraldehyde cacodylate buffer, and the subsequent post-fixation was done in osmium tetroxide. Ultrathin sections of Epon resin blocks of each sample (50–60 nm) were collected on copper grids, stained with uranyl acetate and lead citrate, and finally examined under a transmission electron microscope (JEM1400, Peabody, MA, USA) with a 12,000 and 25,000× magnification.

### 4.9. RNA Isolation

An ~8 mg biopsy previously stored in RNA (Qiagen Redwood City, CA, USA) at −80 °C was used to isolate total RNA through TriFastTM protocol (peQlab, VWR Company, Radnor, PA, USA). The total RNA extracted was treated with RNase-free DNAse I (Qiagen Redwood City, CA, USA).

#### 4.9.1. RNA Preparation for Sequencing

Immediately after Total RNA DNAse treatment of LV sample, long RNA (>200 bp, mRNA) and small RNA (<200 bp, miR) were separated using different ethanol percentage solution and Qiagen mini Kit columns. When necessary, samples were cleaned up using the Qiagen RNeasy MinElute Cleanup Kit to remove genomic DNA traces. RNA integrity and quality (RIN > 8.0) were acquired using Agilent Technologies 2100 Bioanalyzer, namely using RNA-specific chip (Agilent Eukaryote Total RNA Nano 1000). To accurate RNA and complementary DNA (cDNA) libraries concentrations, Qubit was used. Importantly, the LV samples used for RNA studies were from animals whose morpho-functional characteristics (echocardiography and histology data) are described in the results section. Also, when possible, the Long and Small RNA enriched fractions used for RNA NGS were isolated from the same LV sample. However, in some samples, the quality of Long RNA or Small RNA isolated did not pass the quality test to enter NGS studies (Table S2).

#### 4.9.2. Bulk mRNA-Sequencing

The long RNA samples (SHAM, *n* = 7; BA, *n* = 6; DEB, *n* = 7) were processed with the Illumina TruSeq Stranded Total RNA Library Prep Kit with Ribo-Zero Gold following the manufacturer’s recommendations. Indexed libraries were pooled and sequenced on the Illumina Hiseq 3000 (paired-end 150 bp) using sequencing-by-synthesis chemistry v4 according to the manufacturer’s protocols. Each TruSeq RNA library produced an average yield of 3.6 Gb of sequencing data, with an average of >80% of the reads achieving a quality score equal to or greater than Q30. R Bioconductor Software was used for the analysis and comprehension of the data. The DEGs were identified by adjusting *p*-value for multiple testing using Benjamini–Hochberg correction with false discovery rate (FDR) ≤0.05 and Log2 fold change (Log2FC), |Log2 FC| ≥ 0.5.

#### 4.9.3. Gene Set Enrichment Analysis

Gene set enrichment analysis (GSEA) was performed using the Kyoto Encyclopedia of Genes and Genomes (KEGG) pathways dataset. A maximum estimate score (MES) was calculated for each query pathway (detailed information in supplementary data). MES value represents a pathway level, where a positive MES value indicates up-enrichment (up-regulation), whereas a negative MES value indicates down-enrichment (down-regulation) of a pathway. For a more detailed information please check supplementaty methods.

Activation/inhibition pathway analysis: Ingenuity pathway analysis (IPA; Qiagen, Redwood City, CA, USA) was used to calculate the activation Z-score that leverages knowledge about the direction of effects, activation/inhibition of a pathway. A differentially expressed genes list (FDR < 0.05 and |Log2FC| ≥ 0.5) was used to perform this analysis. IPA uses up- and down-regulation of genes to predict Z-score, so gene lists were not separated by Log2FC. Pathways with –Log(*p*-value) ≥1.3 were considered significantly enriched pathways (Fisher’s exact test *p*-value). A Z-score ≥1.5 predicts pathway activation, and a Z-score ≤1.5 predicts its inhibition in that specific comparison.

### 4.10. microRNA-Sequencing

The small RNA samples (SHAM, *n* = 5; BA, *n* = 8; DEB, *n* = 6) were processed with Illumina’s TruSeq Small RNA protocol. MicroRNAs were size-selected on an 8% polyacrylamide gel, purified, quantified, and pooled for multiplexed sequencing on the Illumina Hiseq 3000 (single end 50 bp) using sequencing-by-synthesis chemistry v4 according to the manufacturer’s protocols. Each TruSeq RNA library produced an average yield of 3.6 Gb of sequencing data, with an average of >80% of the reads achieving a quality score equal to or greater than Q30. The differentially expressed miRs were identified by adjusting the *p*-value for multiple testing using Benjamini–Hochberg correction with FDR ≤0.05 and |Log2FC| ≥ 1.

A real-time polymerase chain reaction (RT-PCR) (Figure S3) was used to validate some of the miRs dysregulated in DEB1 compared to DEB2. For this exploratory analysis, we used total RNA isolated from new samples and total RNA extracted for NGS experiments. Detailed information in Supplementary methods (Table S3).

### 4.11. mRNA-miR Integrative Analysis

IPA microRNA target filter analysis was used to perform mRNA-miR integrative analysis. List of dysregulated miR and mRNA were combined in IPA software, and different filters were used to obtain the final list of miR-mRNA interactions: (a) miR with Log2FC ≥ 1; (b) mRNA with Log2FC ≤ 0.5; (c) confidence level equal to “moderate”, “highly predicted” and “experimentally observed”. We chose these filters based on miR’s primary biological role, mRNA translation inhibition, or degradation. Gene ontology analysis was performed using the DAVID bioinformatics database [61] and the IPA gene view. Pathway analysis of final predicted targets was performed using IPA core analysis.

### 4.12. Statistical Analysis

Statistical analysis was performed using GraphPad Prism (version 7.0. www.graphpad.com). D’Agostino & Pearson normality test and Shapiro–Wilk normality test were run to test variables normal distribution. For normally distributed variables, we performed a one-way analysis of variance (ANOVA) test followed by Holm–Sidak multiple comparisons test to assess differences between groups. For data distribution that fails normality, we used the non-parametric Kruskal–Wallis test, followed by the adequate multiple comparisons test. For the correlations test, we used the Pearson correlation for variables following Gaussian distribution. The mixed-effects ANOVA test was used for echocardiographic data measured at different time points. Group data are presented as means ± SEM or as median (interquartile range). Statistical significance was set at a *p*-value < 0.05.

## Figures and Tables

**Table 1:** Terminal echocardiographic, hemodynamic, and morphometric data for SHAM, BA, DEB1, and DEB2 groups.

Morphometry	Parameters	SHAM (*n* = 20)	BA (*n* = 12)	DEB1 (*n* = 5)	DEB2 (*n* = 5)
	TL, cm	1.79 ± 0.02	1.75 ± 0.02	1.80 ± 0.05	1.77 ± 0.02
	Heart/TL, g/cm	70.39 ± 1.72	89.96 ± 7.14 ^α^	91.16 ± 5.04 ^α^	70.45 ± 2.14 ^χ δ^
	LV + Sp/TL, g/cm	44.67 ± 2.29	60.03 ± 4.91 ^α^	57.01 ± 4.61	44.17 ± 2.83
	Lungs/TL, g/cm	91.25 ± 5.00	99.20 ± 7.88	105.30 ± 10.23	92.28 ± 7.16
Echocardiography	LV mass, mg	99.6 ± 5.5	151.4 ± 9.6 ^ααα^	110.7 ± 13.4	113.0 ± 9.7
	LVEF, %	68.55 ± 1.92	70.07 ± 3.46	72.81 ± 4.06	61.31 ± 4.59
	EDV, uL	65.34 ± 3.26	65.26 ± 4.88	62.97 ± 11.92	66.91 ± 4.47
	IVRT, ms	18.88 ± 1.05	17.03 ± 1.56	13.47 ± 1.18	19.32 ± 2.50
	E/e’	13.57 ± 0.66	20.28 ± 1.94 ^αα^	19.27 ± 1.28 ^α^	14.07 ± 0.68
	LAA, cm^2^	0.040 [0.039; 0.043]	0.064 [0.040; 0.086]	0.068 [0.060; 0.077]	0.043 [0.041; 0.044]
	HR, bpm	451.1 ± 22.6	434.8 ± 12.96	516.1 ± 19.11	492.5 ± 41.70
Haemodynamic	LVESP, mmHg	80.70 ± 3.42	108.10 ± 8.84 ^α^	89.19 ± 3.67	87.69 ± 8.85
	LVEDP, mmHg	4.75 ± 0.67	10.71 ± 2.47 ^α^	3.28 ± 0.93 ^χ^	4.06 ± 0.68

Animals presenting with an aortic velocity below 2.5 m/s seven weeks after aorta constriction were excluded from the study. E/e’, the ratio of mitral peak velocity of the early filling (E) to early diastolic mitral annular velocity (e’); IVRT, isovolumetric relaxation time; LVEF, left ventricle ejection fraction; EDV, end-diastolic volume; HR, heart rate; LAA, left atrium area; LV, left ventricle; LV + Sp, left ventricle + septum; LV mass, left ventricle mass; LVEDP, left ventricle end-diastolic pressure; LVESP, left ventricle end-systolic pressure; TL, Tibia length. Values are represented by mean ± SEM. For variable LAA values are represented by median [minimum; maximum] and number per group is (SHAM, *n* = 3; BA, *n* = 4; DEB1, *n* = 2; DEB2, *n* = 2). One-way-ANOVA followed by Holm-Sidak’s test for variables: Heart/TL, LVEF and TL; Kruskal-Wallis test for variables: E/e’, EDV, HR, IVRT, LAA, Lungs/TL, LV mass, LVEDP, LVESP and LV + Sp/TL. vs. SHAM: ^α^, *p* < 0.05; ^αα^, *p* < 0.01; ^ααα^, *p* < 0.001; vs. BA: ^χ^, *p* < 0.05; vs. DEB1: ^δ^, *p* < 0.05.

## Supplementary Materials

The following are available online at https://www.mdpi.com/1422-0067/21/24/9687/s1, Figure S1: Representative images of echo-Doppler and tissue Doppler echocardiography; Figure S2: Representative transmission electron micrographs (TEMs; 12,000×) from left ventricle; Figure S3: Canonical pathways activation Z-score analysis in BA vs SHAM; Figure S4: Real Time-PCR for miRs dysregulated between DEB1 and DEB2; Figure S5: Top down-regulated mRNA and its respective miR in BA vs SHA; Figure S6: Overlapping of the ten most significant canonical pathway analysis based on the list of predicted targets in BA compared to SHAM; Figure S7: Upstream Regulators analysis; Figure S8: Canonical pathways activation Z-score analysis in for the different comparations. Table S1: Phenotypic characterization of a group of animals with nine weeks of banding; Table S2: Table with samples information entering in Next Generation Sequence studies; Table S3: List of miRNAs quantified by RT-PCR. Data S1:List of differentially-expressed transcripts between groups and Gene Ontology for the top genes found dysregulated in all groups; Data S2: IPA Z-score analysis based on differentially-expressed genes for all comparations; Data S3:List of dysregulated miRs between groups; Data S4: miR-mRNA Functional Annotation; Data S5: Gene enrichment analysis after miR-mRNA integrative analysis. Supplementary Methods.
