# Supplementary material for: The Degree of Cardiac Remodelling before Overload Relief Triggers Different Transcriptome and miRome Signatures during Reverse Remodelling (RR)—Molecular Signature Differ with the Extent of RR
Source: Int J Mol Sci. 2020 Dec 18;21(24):9687. doi: 10.3390/ijms21249687 (PMC7766898; doi:10.3390/ijms21249687)
Supplement: Supplementary file 1 [file ijms-21-09687-s001.pdf]

<u>**Supplementary Data**</u>

Supplementary Tables and Figures

Supplement tables:

Table S1: Phenotypic characterization of a group of animals with nine weeks of banding.

	Parameters	SHAM (n=20)	BA7 (n=13)	BA9 (n=11)
Morphometry	TL, cm	1.79±0.02	1.75±0.02	1.74±0.02
	Heart/TL, g/cm	70.39±1.72	89.96±7.14 $^{\alpha}$	103.80±9.79 $^{\alpha\alpha\alpha\alpha}$
	LV+Sp/TL, g/cm	44.67±2.29	60.03±4.91$^{\alpha}$	68.65±6.06 $^{\alpha\alpha}$
	Lungs/TL, g/cm	91.25±5.00	99.20±7.88	116.00±18.16
Echocardiography	LV mass, mg	99.6±5.5	151.4±9.6 $^{\alpha\alpha\alpha}$	148.2±14.34$^{\alpha\alpha\alpha}$
	LVEF, %	68.55±1.92	70.07±3.46	51.35±3.40 $^{\alpha\alpha\alpha\ \chi\chi\chi}$
	EDV, ul	65.34±3.26	65.26±4.88	85.53±5.05 $^{\alpha\alpha\chi\chi}$
	IVRT, ms	18.88±1.05	17.03±1.56	16.76±2.61
	E/e´	13.57±0.66	20.28±1.94 $^{\alpha\alpha}$	19.97±1.37 $^{\alpha\alpha}$
	LAA, cm^2	0.040[0.039;0.043]	0.064[0.040;0.086]	0.072[0.049;0.081]
Haemodynamic	LVESP, mmHg	80.70±3.42	108.10±8.84 $^{\alpha}$	141.1±15.01$^{\alpha\alpha\alpha\chi}$
	LVEDP, mmHg	4.75±0.67	10.71±2.47 $^{\alpha}$	11.74±6.71
	HR, bpm	491.8±22.8	457.6±26.5	434.5±27.8
Histology	Fibrosis, %	11.17±1.19	22.67±3.59 $^{\alpha\alpha}$	37.47±3.46 $^{\alpha\alpha\alpha\chi\chi}$

E/e', the ratio of mitral peak velocity of the early filling (E) to early diastolic mitral annular velocity (e'); IVRT, isovolumetric relaxation time; LVEF, left ventricle ejection fraction; EDV, end-diastolic volume; HR, heart rate; LAA, left atrium area; LV, left ventricle; LV+Sp, left ventricle + septum; LV mass, left ventricle mass; LVEDP, left ventricle end-diastolic pressure; LVESP, left ventricle end-systolic pressure; TL, Tibia length. Mean ± SEM represents values. Variable LAA values are represented by median [minimum; maximum] and number per group is (SHAM, n=3; BA7, n=4; BA9, n=6). One-way-ANOVA followed by Holm-Sidak's test for variables: E/e', HR, EDV, LVEF, LVESP, and LV mass; Kruskal-Wallis test for variables: Heart/TL, IVRT, LAA, Lungs/TL, LVEDP, and LV+Sp/TL. vs SHAM: α, $P<0.05$; αα, $P<0.01$; ααα, $P<0.001$; vs BA: χ, $P<0.05$; χ χ, $P<0.01$.

Table S2: Table with samples information entering in Next Generation Sequence studies.

Group	Long RNA	Small RNA
SHAM	8S1, 8S5, 9S1, 9S3, 8S3, 8S4, 9S4	8S1, 8S5, 9S1, 8S4, 9S4
BA7	6B12, 6B9, 8B2,9B2,9B5,9B7	6B5, 6B9, 8B1, 8B2, 9B1, 9B2, 9B5, 9B7
DEB1	8B11, 8B12, 9B6	6B2, 8B11, 8B12
DEB2	7B4, 9B3, 9B8, 9B11	7B4, 9B8, 9B11

Table S3: List of miRNAs quantified by RT-PCR with the respective Applied Byosystems assay IDs.

MiRNA assay name	Assay ID
mmu-miR-208b-3p	mmu482934_mir
hsa-miR-199a-5p	478231_mir
mmu-miR-199a-3p	mmu480983_mir
mmu-miR-214-3p	mmu481652_mir
hsa-miR-34c-5p	478052_mir

Supplement figures:

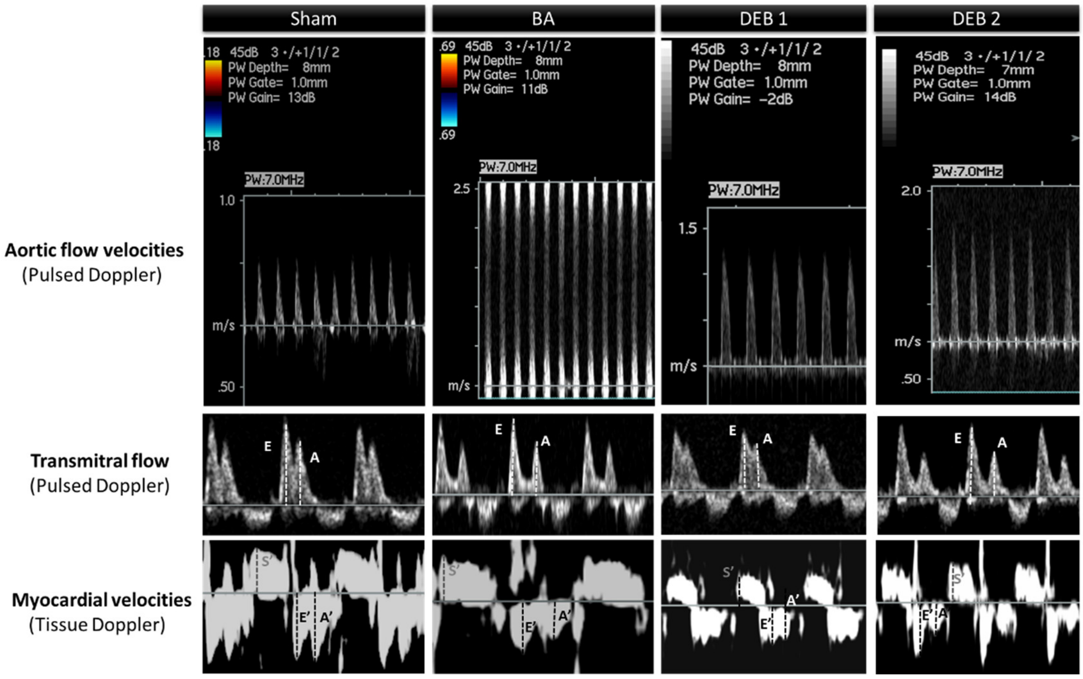

Figure S1: Representative images of echo-Doppler and tissue Doppler echocardiography for all groups.

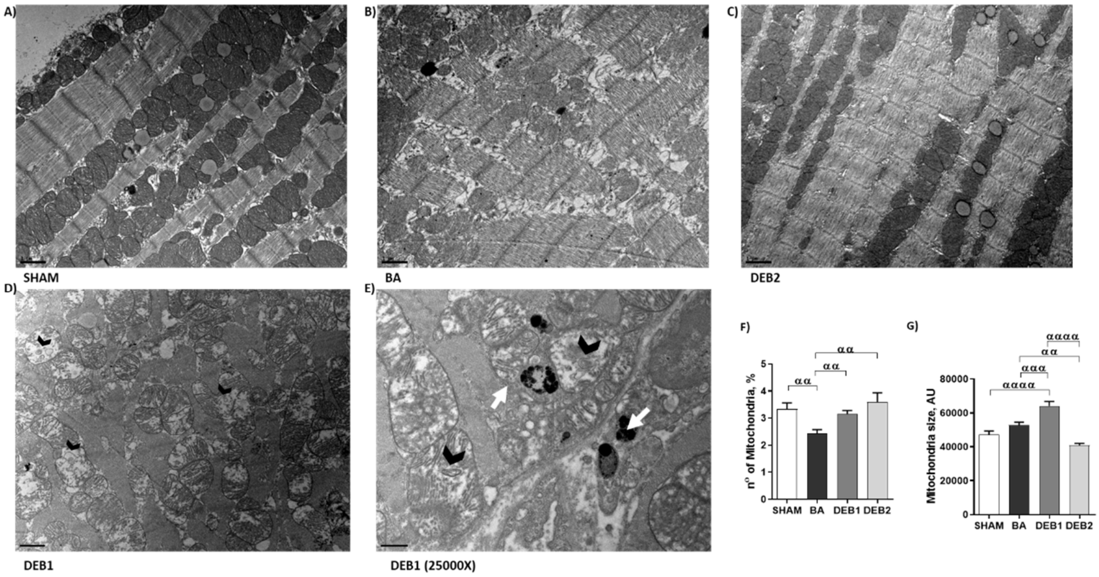

Figure S2: Representative transmission electron micrographs (TEMs; 12000x) from left ventricle of SHAM (A), BA (B), DEB2 (C) and DEB1 (D). Image with bigger magnification (25000x) of DEB1 tissue showing mitochondria swelling (whitespaces inside mitochondria), vacuole-like structures (blackhead arrow), unknown material accumulation (white arrow) (E). Number (F) and area (G) of mitochondria in all groups (15 images per animal were analyzed, n=3 animals). Representative images from each group. Values are represented as mean ± SEM. Kruskal-Wallis test. αα, P<0.01; ααα, P<0.001; αααα, P<0.0001.

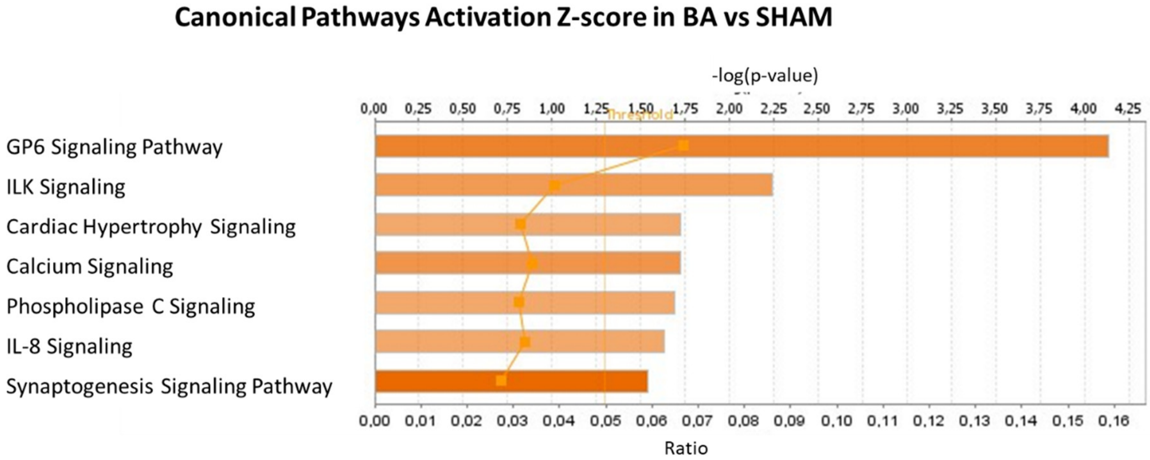

Figure S3: Canonical pathways activation Z-score analysis in BA vs SHAM. Pathways with Z-score ≥1.5 were predicted to be active (orange bars), and pathways with Z-score ≤1.5 were predicted to be inhibited (blue bar). The threshold for significance was –Log(p-value) ≥1.3 (Threshold line) (Fisher's Exact test p-value). The orange line represents the ratio between Up-regulated and down-regulated transcripts. Analysis performed using Ingenuity Pathways Analysis Software, Qiagen.

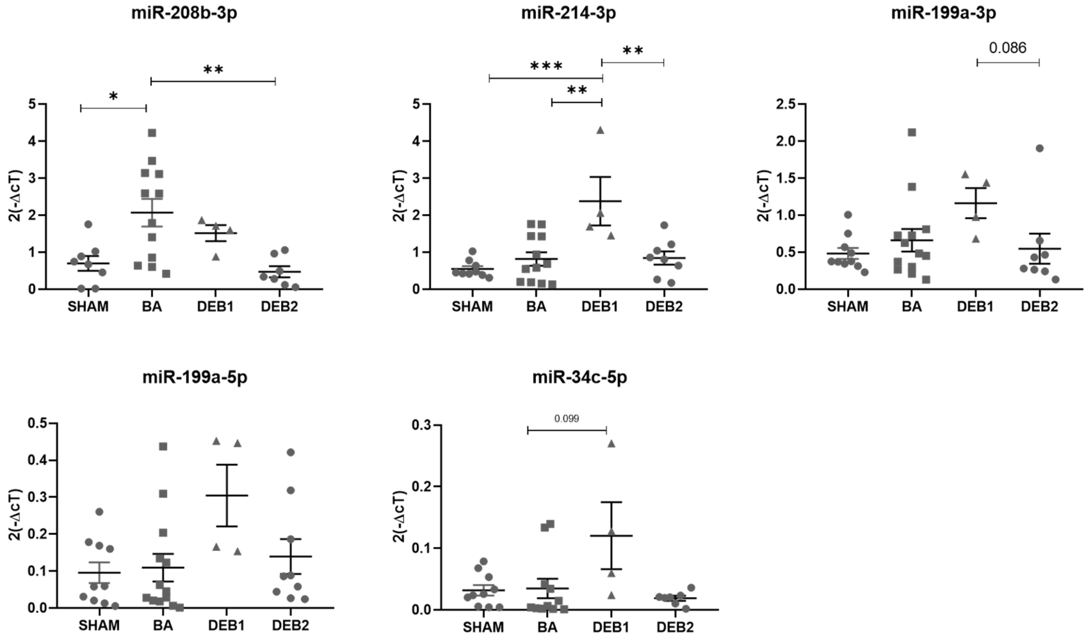

Figure S4: Real Time-PCR for miRs dysregulated between DEB1 and DEB2.

Top down-regulated mRNA and it´s respective miR in BA vs SHAM

miR ID	Log2FC	Target symbol	Target Name	Log2 FC	Confidence
		NFIL3	Nuclear factor. interleukin 3. regulated	-1.357	Moderate (predicted)
miR-199b-5p	2.009	NPAS2	Neuronal PAS domain protein 2	-1.767	Moderate (predicted)
		CDKN1A	Cyclin dependent kinase inhibitor 1A	-1.767	Experimentally Observed
		DDIT4	DNA damage inducible transcript 4	-1.084	Moderate (predicted)
miR-32-5p	1.748	HAS2	Hyaluronan synthase 2	-1.452	Moderate (predicted)
		DDIT4	DNA damage inducible transcript 4	-1.084	High (predicted)
		ELN	Elastin	-1.140	Moderate (predicted)
miR-101a-3p	1.127	HAS2	Hyaluronan synthase 2	-1.452	Moderate (predicted)
miR-455-5p	1.102	HAS2	Hyaluronan synthase 2	-1.452	Moderate (predicted)

Figure S5: Top down-regulated mRNA and its respective miR in BA vs SHAM. Ingenuity pathway analysis software, Qiagen.

Gene-term Enrichment analysis for down-regulated mRNAs that are targets for the top up-regulated miRs in BA vs SHAM

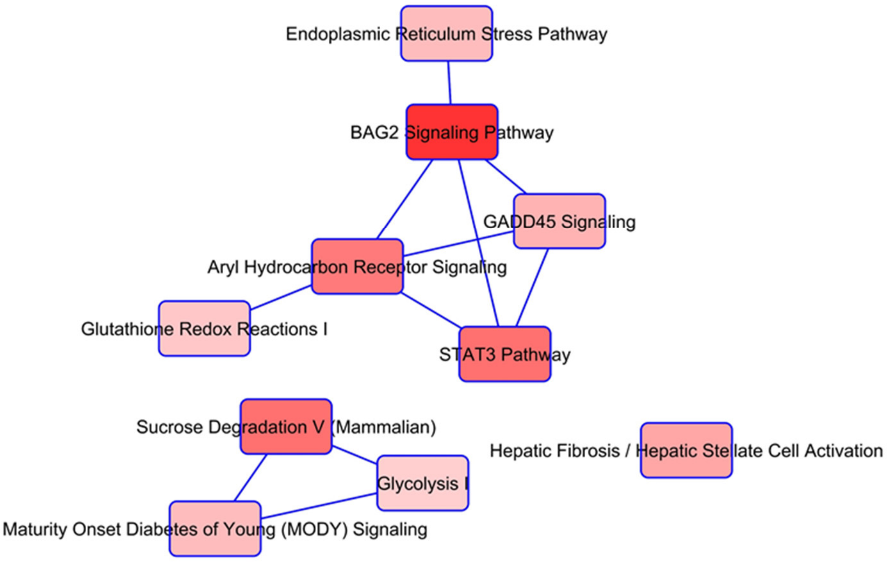

Figure S6: Overlapping of the ten most significant canonical pathway analysis based on the list of predicted targets in BA compared to SHAM. Ingenuity pathway analysis software allowed to combine data set from miR and mRNA seq, aiming to discover a potential relationship between these two biological molecules and its role in reverse cardiac remodeling. Since the main biological role of miR is mRNA translation inhibition or degradation, the data set was filtered to show only relationships with miR up-regulated (Log2 FC≥1) and mRNA downregulated (Log2 FC≤-0.5). The network of overlapping canonical pathways shows each pathway as a single "node" colored proportionally to the Fisher's Exact Test p-value, where brighter red is equal to more significant. A line connects any two pathways when at least one data set is shared between them.

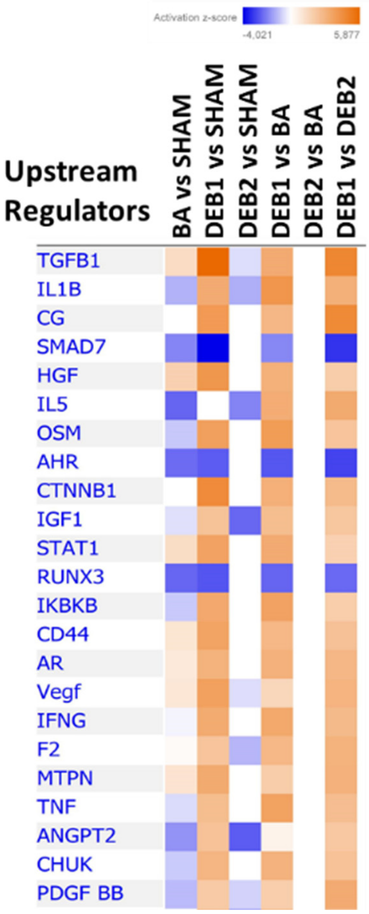

Figure S7: Upstream Regulators analysis showing the top candidates for BA, DEB1 and DEB2 vs SHAM, DEB1, and DEB2 vs BA and DEB1 vs DEB2. Regulators with Z-score ≥2.0 were predicted to be active (orange), and a Z-score ≤2.0 indicates inhibition (blue). Analysis performed using Ingenuity Pathways Analysis Software, Qiagen

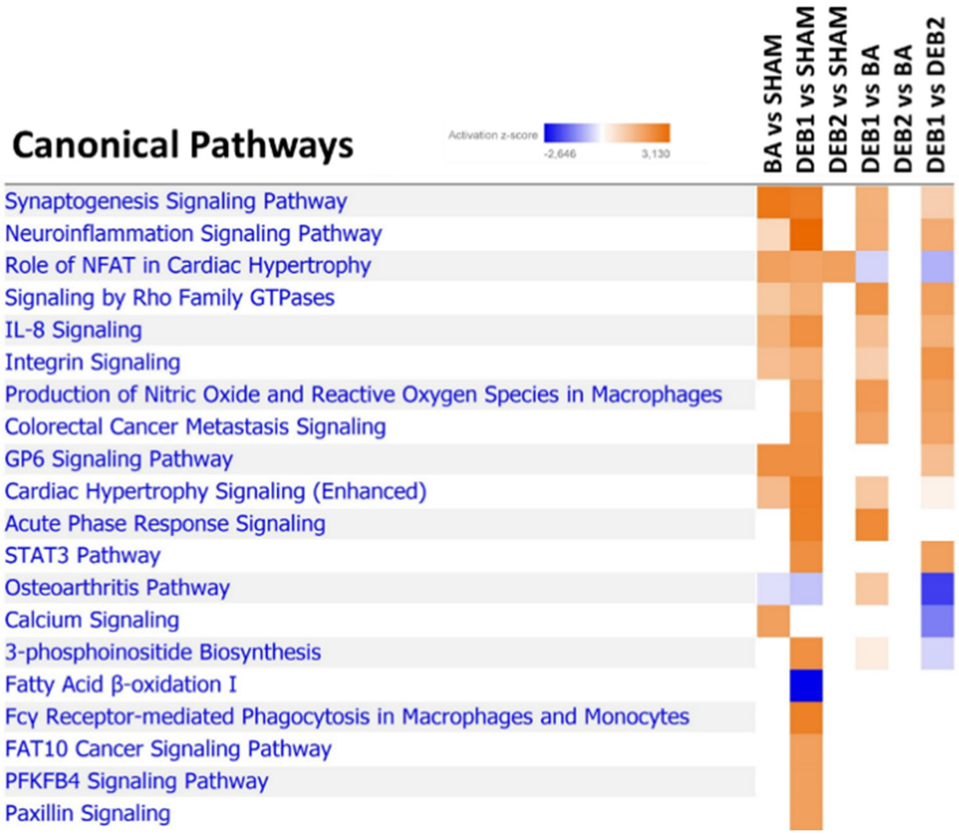

Figure S8: Canonical pathways activation Z-score analysis in for the different comparations. Pathways with Z-score ≥1.5 were predicted to be active (orange bars), and pathways with Z-score ≤1.5 were predicted to be inhibited (blue bar). The threshold for significance was –Log(p-value) ≥1.3 (Threshold line) (Fisher's Exact Test p-value). The orange line represents the ratio between Up-regulated and down-regulated transcripts. Analysis performed using Ingenuity Pathways Analysis Software, Qiagen

Supplementary Methods

Bulk mRNA-Seq raw data analysis:

All raw sequence reads available in FastQ format were mapped to the mouse genome (mm10) using Tophat2 with Bowtie2 option [62,63], where adaptor sequences were removed using trim galore before read mapping. BAM files containing the alignment results were sorted according to the mapping position. Raw read counts for each gene were calculated using featureCounts from Subread package [64], and DEseq2 was used to perform the analysis of differential gene expression, where genes with raw counts as input [65].

Gene Set Enrichment description:

Gene Set Enrichment Analysis (GSEA) [66] was performed using the Kyoto Encyclopedia of Genes and Genomes (KEGG) pathways dataset. First, genes were ranked decreasingly according to the Log2FC of expression. For each query pathway, if gene *i* is a member of the pathway, it is defined as

$$Xi = \sqrt[2]{\frac{N-G}{G}}$$

If gene *i* is not a member of the pathway, it is defined as

$$X_i = -\sqrt[2]{\frac{G}{N-G}}$$

where N indicates the total number of genes and G indicates the number of genes in the query pathway. Next, a max running sum across all N genes Maximum Estimate Score (MES) is calculated as

$$MES = \max_{1 \leq j \leq N} \sum_{i=1}^{j} Xi$$

The permutation test was performed 500 times to judge MES values' significance, where the null hypothesis is that the pathway is not enriched in the ranking. If the query pathway with a nominal p-value less than 0.05 and adjusted p-value for multiple testing using Benjamini-Hochberg correction with FDR values less than 0.1, the null hypothesis would be rejected, and the query pathway would be significantly enriched.

microRNA-Seq raw data analysis:

All raw sequence reads were obtained in FastQ format. Adaptor sequences were removed using trim_galore before read mapping. Bowtie2 was used to map the sequence to the mouse mature miR sequence obtained from miRBase database [63,67], where reads with seed sequence no shorter than 20bps with 0 mismatch and less than or equal 5 multiple matching positions are successfully aligned. As bowtie1 has a higher incorrect mapping rate for miR, here we decide to use bowtie2 [68]. BAM files containing the alignment results were sorted according to the mapping position. Raw read counts for each miRs were calculated using Samtools with idxstats function [69]. DEseq2 was used to perform the analysis of differential miR expression, where miR with raw counts as input [65].

MicroRNA quantification by RT-PCR

RNA was isolated from mice left ventricle tissue using the miRNeasy® Micro Kit (Qiagen; Cat no: 217084) according to the manufacturer's instructions. RNA quantitation and purity were evaluated by spectrophotometric analysis (NanoDrop 2000c, ThermoFisher Scientific, USA). Ten ng of extracted RNA was converted to cDNA with the TaqMan™ Advanced MicroRNA cDNA synthesis Kit (Applied Biosystems; Cat no: A28007), per the manufacturer's instructions, in a T100™ Thermal Cycler (Bio-Rad) thermocycler. Quantitative RT-PCR was performed with Taqman™ Advanced MicroRNA assays (Applied Biosystems; Cat no: A25576) (Table S3) and TaqMan™ Fast Advanced Master Mix (Applied Biosystems; Cat no: 4444964), in a Thermo Scientific™ PikoReal™ Real-Time PCR System (96-wells). Each sample was analyzed in triplicate, and the average Cycle Threshold (Ct) was used. Relative expression was calculated with the 2-ΔΔCT method [70]. Reference gene selection was based on NGS expression data of a group of Taqman™ Advanced MicroRNA assays described as good endogenous controls. The hsa-miR-186-5p Taqman™ Advanced MicroRNA (Assay ID: 002285) presented the most stability across all samples (data not shown) and was, therefore, used as the endogenous control.

"Non-standard Abbreviations and Acronyms."

AS, aortic stenosis; **AVR,** aortic valve replacement; **cDNA**, complementary DNA; **DAMPs**, damage-associated molecular pathways; **DEGs**, differentially-expressed genes; **E/e',** ratio of mitral peak velocity of early filling (E) to early diastolic mitral annular velocity (e'); **ECM**, extracellular matrix; **LVEF**, left ventricle ejection fraction; **FDR**, false discovery rate; **GSEA**, gene set enrichment analysis; **HF,** heart failure; **IPA**, ingenuity pathway analysis; **IVS,** interventricular septum; **LAA**, left atrium area; **LV,** left ventricle; **LVD,** left ventricle diameter; **LVPW,** left ventricle posterior wall; **LVAD,** left ventricle assist devices; **Log2FC,** Log2 Fold Change; **LVEDP**, left ventricle end-diastolic pressure; **LVESP**, left ventricle end-systolic pressure; **KEGG,** Kyoto Encyclopedia of Genes and Genomes; **MES**, maximum estimate score; **miRs,** microRNAs; **mRNA,** messenger RNA; **Bulk mRNA-Seq,** mRNA sequencing; **NAD^+**, nicotinamide adenine dinucleotide; **NLRs**, Nod-like receptors; **PCr/ATP**, phosphocreatine/adenosie triphosphate ratio;

RR, reverse remodelling; **Small-Seq,** small RNA sequencing; **TAC**, trasverse aortic constriction; **TCA cycle**, citrate cycle; **TLRs**, Toll-like receptors;

Genes list mention in text: ***Acsm5,*** acyl-CoA synthetase medium-chain family member 5***; Acot1,*** acyl-CoA thioesterase 1*;* ***Angpt1*****,** angiopoietin 1; ***Angptl7***, angiopoietin-like 7; ***Arntl***, aryl hydrocarbon receptor nuclear translocator-like; ***Atp2a1***, sarcoplasmic/endoplasmic reticulum calcium ATPase 1; ***BCCA***, branch chain amino acids; ***Camkii,*** calcium/calmodulin-dependent protein kinase II; ***Chil1***, chitinase-like 1; ***Cited4***, Cbp/p300-interacting transactivator 4; ***Cilp***, cartilage intermediate layer protein; ***Col8a2***, collagen, type VIII, alpha 2; ***Col12a1***, collagen, type XI, alpha 1; ***Comp***, cartilage oligomeric matrix protein; ***Crlf1***, cytokine receptor-like factor 1; ***Ddc,*** dopa decarboxylase; ***Ddit4***, DNA damage-inducible transcript 4; ***Dtl***, denticleless E3 ubiquitin protein ligase; ***Eln***, elastin; ***Fgf16***, fibroblast growth factor 16; ***Fmod***, fibromodulin; ***Gadd45g,*** growth arrest and DNA-damage-inducible protein; ***Gdf15***, growth differentiation factor 15; ***Gsk3β***; glycogen synthase kinase 3; ***Has2***, hyaluronan synthase 2; ***Hs3st5***, heparan sulfate-glucosamine 3-sulfotransferase 5, ***ILK***, integrin linked kinase; ***IL-6***, interleukin-6; ***IL-8***, interleukin-8; ***Lox***, lysyl oxidase; ***Ltbp2***, latent transforming growth factor beta binding protein 2; ***Klhl23***, kelch like family member 23; ***Mmp***, matrix metalloproteinase; ***Mmp3***, matrix metalloproteinase 3; ***Myh7***, myosin heavy polypeptide 7 (beta); ***Myl4***, myosin light polypeptide 4; ***Myl7***, myosin light polypeptide 7; ***NFAT***, nuclear factor of activated T-cells; ***Nfil3***, nuclear factor interleukin 3; ***Nox4***, NADPH oxidase 4; ***Npas2***, neuronal PAS domain protein 2; ***Omd***, osteomodulin; ***Per2***, period circadian clock 2; ***Per3***, period circadian clock 3; ***Pl15***, peptidase inhibitor 15; ***Postn***, periostin; ***Rcan1***, regulator of calcineurin 1; ***Rhobtb1***, Rho-related BTB domain containing 1; ***Sfrp2***, secreted frizzled-related protein 2; ***Sln***, sarcolipin; ***Spon2***, spondin 2; ***Tef***, thyrotroph embryonic factor; ***Tgf-β1***, transforming growth factor beta 1; ***Tgf-β3***, transforming growth factor beta 3; ***Thbs4***, thrombospondin 4; ***Timp1***, tissue inhibitor of metalloproteinase 1; ***Tnf-α***, tumor necrosis factot alpha; ***Vegfd***, vascular endothelial growth factor D;
